# Mapping Proton‐Coupled Electron Transfer With Real Space Coordinates

**DOI:** 10.1002/anie.5050035

**Published:** 2026-07-23

**Authors:** Adam Šrut, Martin Diefenbach, Marvin L. Kronenberger, Benjamin J. Lear, Vera Krewald

**Affiliations:** ^1^ Department of Chemistry Quantum Chemistry TU Darmstadt Darmstadt Germany; ^2^ Department of Chemistry The Pennsylvania State University Pennsylvania PA USA

**Keywords:** computational chemistry, electron transfer, nuclear coordinates, proton transfer, proton‐coupled electron transfer

## Abstract

Both electron transfer (ET) and proton transfer (PT) are common steps in energy conversion across chemistry and biology. Coupling of these two transfer events reduces the energy demand relative to either individual process, but introduces a fundamentally new process—proton‐coupled electron transfer (PCET)—with its own demands for a suitable theoretical description. Conceptualization of PCET usually involves a square scheme representing ET, PT, and PCET steps, each generating states with different energies. While intuitive, these square schemes do not offer structural insights such as the identities and contributions of nuclear motions to PCET. Herein, we present a computational approach that maps these square schemes onto real space coordinates (Å), from which ground and excited‐state potential energy surfaces can be generated. This mapping involves the identification of PT and ET coordinates and reconstruction of the potential energy surfaces in orthogonalized coordinates. We find qualitative differences in key features of the surfaces for two distinct processes within PCET, namely concerted proton–electron transfer and hydrogen atom transfer, which may help to distinguish different PCET scenarios.

## Graphical abstract

We explore how proton‐coupled electron transfer (PCET) proceeds on a potential energy surface defined by two coordinates for proton and electron transfer that are obtained from quantum chemical calculations. The two‐dimensional maps for PCET show distinct characteristics for concerted proton–electron transfer and hydrogen atom transfer. Based on these observations, we transfer the conceptual energy‐based square scheme into the real‐space domain.

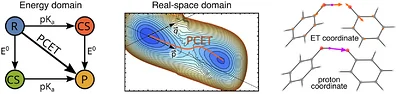

## 1 Introduction

**FIGURE 1:**
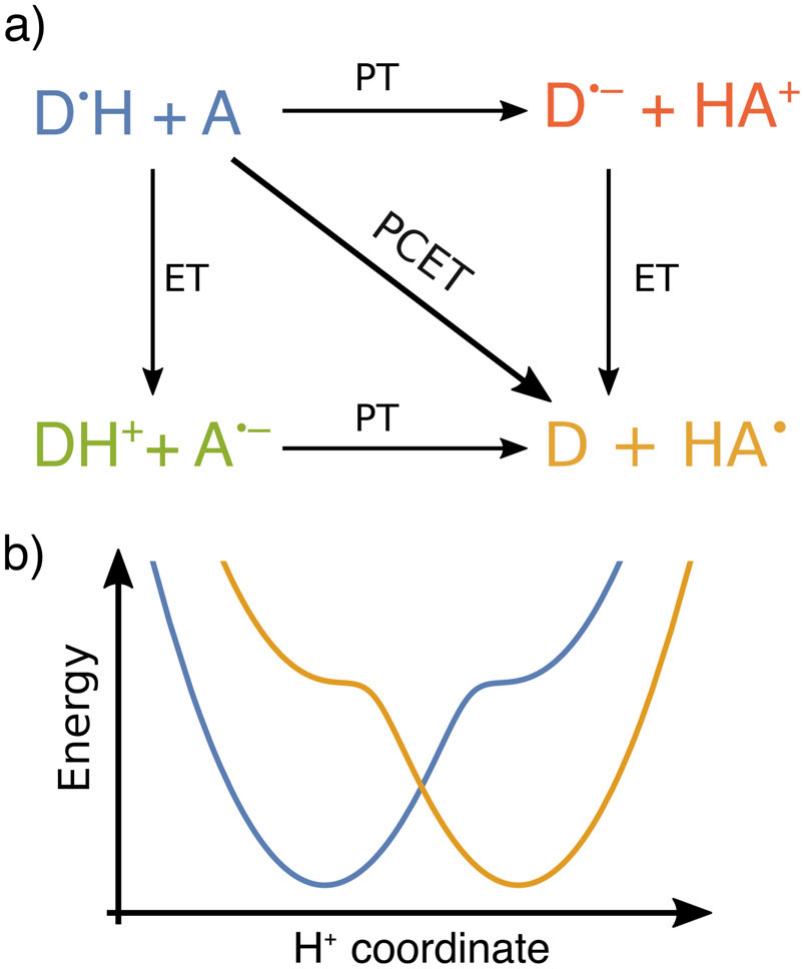
(a) Square scheme for PCET; proton transfer (PT) and electron transfer (ET) between a donor (D) and acceptor (A) are depicted as individual processes on vertical and horizontal lines; PCET is represented by the diagonal arrow. (b) Diabatic curves of reactant and product (top‐left and bottom‐right corners in panel (a)) as functions of a proton coordinate.

Proton‐coupled electron transfer (PCET) is important in many areas of chemistry, biology, and materials science, and has been reviewed extensively over the past decades [1, 2, 3, 4, 5, 6, 7, 8, 9, 10]. PCET is conceptualized in so‐called square schemes, see Figure 1a, where the horizontal axes refer to the isolated proton transfer event, the vertical axes describe the isolated electron transfer event, and the diagonal axis shows the PCET step. The axes are connected to experimentally accessible descriptors, that is, the $pK_{a}$ value and the redox potential, respectively [11], making it clear it is an energy‐based scheme. A chemically intuitive explanation for PCET being preferable over sequential steps is that it avoids the formation of high‐energy, charge‐separated intermediates depicted in the off‐diagonal corners of the square scheme.

The vocabulary used to describe individual steps and subclassifications in PCET varies significantly in the literature [5, 12], and herein we will use the following. Proton transfer (PT) refers to the transfer of a proton in the sense of a pure Brønsted acid–base reaction. Electron transfer (ET) refers to the transfer of an electron in the sense of a redox reaction, which may be intramolecular and is commonly modeled using Marcus–Hush theory [13, 14, 15]. Sequential PT‐ET and ET‐PT reactions require a (meta‐)stable intermediate corresponding to either of the off‐diagonal corners of the square scheme. If both ET and PT proceed via a common transition state and hence a single kinetic step, this is labeled concerted proton–electron transfer (CPET) [11]. Notably, proton and electron can be transferred to or from different sites in a multi‐site process [16, 17]. In contrast, the electron and proton traveling together from donor to acceptor with the electron residing in the H 1s orbital [17] is referred to as hydrogen atom transfer (HAT) [5, 18, 19, 20].

In quantum chemical calculations, it is easy to identify the structures of the molecules in the top‐left and bottom‐right corners of the square scheme; these are well‐defined minima on the potential energy surfaces (PESs) corresponding to reactants and products. Methodologies to identify molecular structures within the square scheme have been developed in extensive efforts, chiefly to decipher the path that a proton will take as it travels from donor to acceptor [21]. In the seminal Hammes–Schiffer model [9, 22, 23], the PCET process can be fully captured by reconstructing ET diabats separated along the proton coordinate, see Figure 1b.

So far, the computational description of PCET has thus relied on reductions of the full PES to the one‐dimensional case shown in Figure 1b. We wondered whether a two‐dimensional model that explicitly defines separate spatial coordinates for the ET and PT processes would provide a more direct connection to the square scheme shown in Figure 1a and thus allow a more finely gradated interpretation of PCET processes, potentially contributing to the question of how to distinguish CPET and HAT raised by Glover and Hammarström [11].

Herein, we propose a novel approach for translating the energy‐based square scheme for PCET into real Cartesian space. We demonstrate this approach using three model systems based on phenol, indole, and toluene, which are prototypical examples of PCET [24, 25, 26, 27, 28]. Our starting point for this endeavor was the realization that the horizontal and vertical axes of the square scheme can never be fully enforced in quantum chemical calculations: the proton and electron motions cannot be completely disentangled if donor and acceptor are described in the same calculation. Separating donor and acceptor into different calculations will neglect their interaction and hence introduce inaccuracies. This raises the question of how to define meaningful paths within the (3N−6) dimensional space that encompass all degrees of freedom relevant to PCET. In the approach proposed herein, we make use of the fact that the Marcus–Hush model is expected to describe the ET part of the PCET process, and define the proton movement as a linear translation from its equilibrium position toward the acceptor atom. The resulting nuclear coordinates can be used to map out the portion of the full (3N−6) dimensional space relevant to the square scheme as a two‐dimensional surface. The path that the system undergoes during the reaction can then be estimated in terms of motions along these coordinates.

## 2 Results

The proposed mapping of the square scheme onto real space coordinates requires clear definitions of both PT and ET nuclear coordinates. We define the proton coordinate, $\vec{p}$, by a linear translation of the proton from its equilibrium position in the optimized geometry of the donor–acceptor entity toward the acceptor atom. We note that other definitions of a proton coordinate are conceivable such as based on the donor‐H stretching mode or resulting from different linear interpolation or fitting schemes, including nonlinear paths. In this first introduction of our proposed two‐dimensional mapping scheme, we will use the linear translation defined above for simplicity.

For the definition of a coordinate that captures the ET process we use the reasoning from Marcus–Hush theory: There is a collective motion of atomic nuclei that brings the system to a configuration where the donor and acceptor orbitals have the same energies and the electron can tunnel between them. We further assume that this collective motion can be described as a single ET coordinate, $\vec{q}$. We have recently developed an approach for quantifying the Marcus–Hush model from *ab initio* calculations [29, 30] where this single coordinate is expressed as a linear combination of normal modes. Hence, we use this approach here to construct the ET coordinate.

**FIGURE 2:**
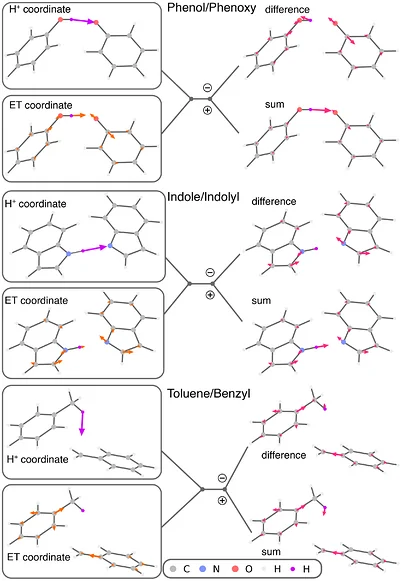
Proton transfer (PT) and electron transfer (ET) coordinates found by linear interpolation and application of the multi‐component fitting procedure, respectively, as described in the text. To obtain orthogonal motions, their sums and differences are computed from their vector representations $\vec{p}$ and $\vec{q}$, which are depicted on the left‐hand side. The lengths of the arrows correspond to the normalized magnitudes of the atomic motions to the normalized coordinate. The transferring proton is highlighted in purple.

These definitions mean that PT and ET nuclear coordinates are well‐defined cuts through the (3N−6)‐dimensional PES (*i.e*., nuclear movements expressed in vector form as linear displacements from the equilibrium geometry, which thus have units of distance (Å)). Figure 2 shows these motions for the studied model systems. A notable feature is that the ET nuclear coordinates of the phenol/phenoxy and indole/indolyl radical pairs contain a substantial movement of the transferring proton. This is a first indication that the PT and ET processes may be coupled.

The vector expression reveals that $\vec{p}$ and $\vec{q}$ are not generally orthogonal, see also Table 1. This is easily verified by visual inspection of Figure 2, where the ET coordinate can contain a significant movement of the transferring proton toward the acceptor atom as noted above. Although $\vec{p}$ and $\vec{q}$ are thus not orthogonal, it is convenient to have orthogonal axes when aiming to construct a two‐dimensional map of the spatial degrees of freedom involved in the overall PCET process. An orthogonal axis frame for this mapping procedure is achieved by taking the sum and the difference of $\vec{p}$ and $\vec{q}$, analogously to taking linear combinations of atomic orbitals to form bonding (positive combination) and antibonding (negative combination) molecular orbitals, see Figure 2.

**TABLE 1:** Key descriptors of the potential energy surfaces spanned by the proton and electron transfer coordinates.

System	Phenol/Phenoxy	Indole/Indolyl	Toluene/Benzyl
TS (eV)^a^	1.49	1.43	3.47
Product minimum (eV)^a^	0.20	0.27	1.79
$2V_{\mathrm{ab}}$ (eV)^b^	0.11	0.15	1.40
$\lambda^{\mathrm{in}}$ (eV)^b^	0.75	0.77	0.81
Reactant minimum^c^	[0, 0]	[0, 0]	[0, 0]
TS^c^	[0.410, 0.061]	[0.482, 0.017]	[1.172, ‐0.001]
Product minimum^c^	[0.762, 0.123]	[0.878, 0.064]	[1.985, 0.030]
$\vec{p}\cdot \vec{q}$ ^d^	0.630	0.630	0.182

^a^ Energetic positions of the transition state (TS) and product minimum relative to the reactant minimum.

^b^ Coupling strength ($V_{\mathrm{ab}}$) and inner‐reorganization energies ($\lambda^{\mathrm{in}}$) in $\vec{q}$ direction.

^c^ Positions of minima and transition states in $\left[\vec{p},\vec{q}\right]$ coordinates (these numbers indicate how much the system must proceed along the depicted $\vec{p}$ and $\vec{q}$ vectors to reach the given point).

^d^ Dot product between $\vec{p}$ and $\vec{q}$ vectors.

The next task is to construct the two‐dimensional PES using the orthogonalized coordinates. This can be done by scanning the region spanned by the orthogonal axes, resulting in a map that contains the reactant and product minima of the square scheme. This portion of the PES now encompasses all nuclear motions associated with proton and electron transfer and provides access to the energy profile of the ground state and any electronically excited states of interest. In reference to the conventional square scheme (Figure 1a), $\vec{p}$ and $\vec{q}$ represent the PT and ET processes, while PCET would be represented by any line or curve connecting the minima of the ground state surface. This lays the foundation to identify the relative positions of reactant and product minima and transition states in terms of motions along $\vec{p}$ and $\vec{q}$ and, in turn, how they are connected to the distinct ET and PT processes. We next consider the chemical model systems.

### 2.1 Phenol/Phenoxy and Indole/Indolyl Radical Pairs: Concerted Proton–Electron Transfer

**FIGURE 3:**
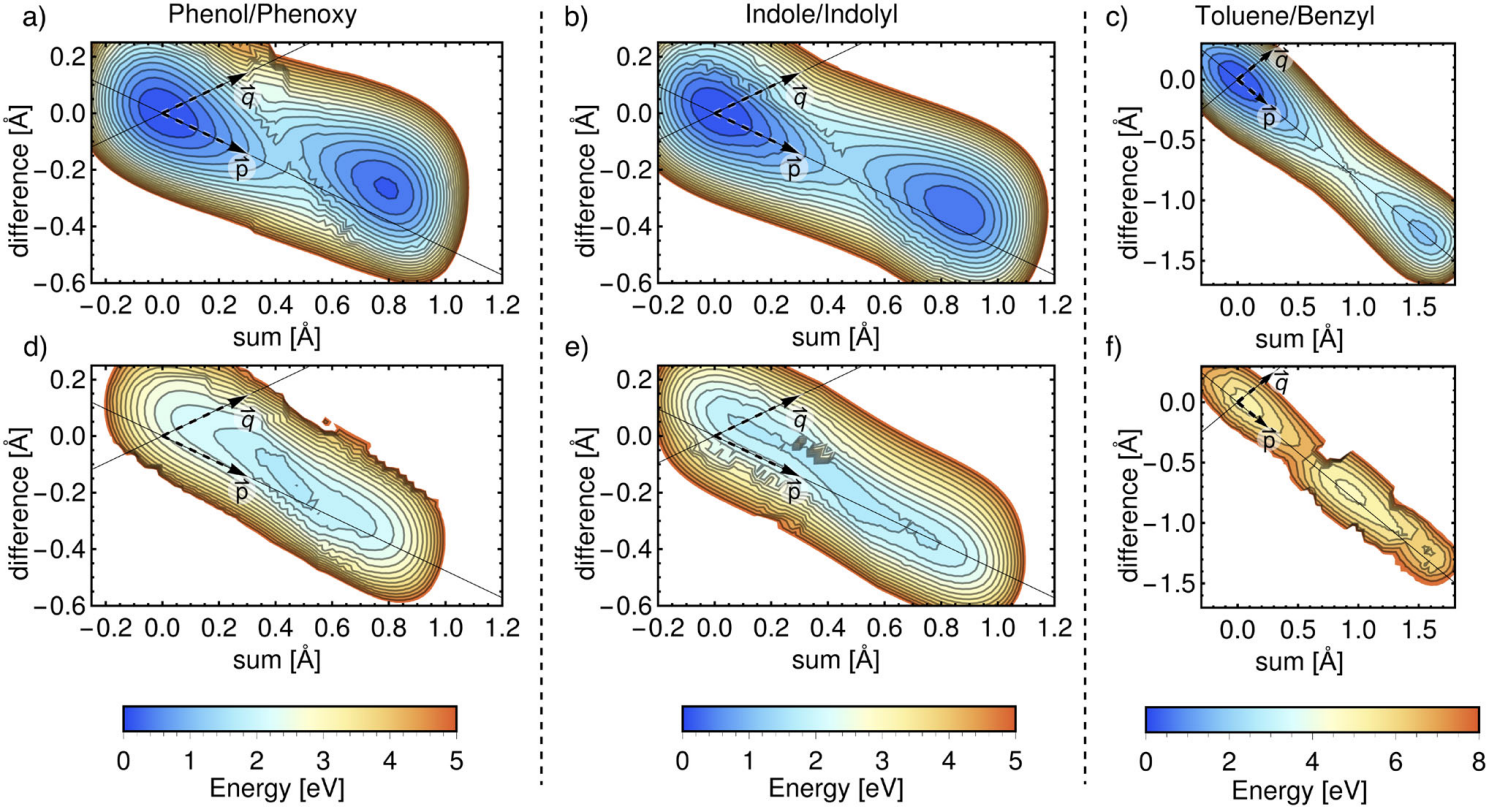
(a–c) Two‐dimensional ground state PESs of the phenol/phenoxy, indole/indolyl, and toluene/benzyl radical pairs spanned by the proton and electron transfer coordinates, respectively. (d–f) Two‐dimensional PESs of the excited ET states with ET character. The *x*‐ and *y*‐axes are taken as the sum and difference of the proton ($\vec{p}$) and electron ($\vec{q}$) transfer coordinates. Contours are spaced by 0.25 eV. The calculations are performed at the CASSCF(3,4)/NEVPT2 level of theory.

The phenol/phenoxy radical pair has been studied extensively with different computational approaches [24, 25, 26, 31]. The consensus is that PCET in this system does not proceed through charge‐separated states, that is, it is a CPET case [24]. The PES with reactant and product minima corresponding to the top‐left and bottom‐right corners of the square scheme are depicted in Figure 3a. Vectors $\vec{p}$ and $\vec{q}$ capture the PT and ET nuclear coordinates, respectively.

We find that the product minimum lies between the $\vec{p}$ and $\vec{q}$ vectors. This observation implies that ET and PT cannot be separated as pure processes: to reach the product minimum, a movement along both is required. More specifically, the product minimum is closer to the axis defined by the proton transfer vector $\vec{p}$, but a substantial movement along the electron transfer vector $\vec{q}$ is needed to reach this minimum as well as the transition state. The coordinates of these points in the basis of $\vec{p}$ and $\vec{q}$ are listed in Table 1. Although the exact reaction pathway is unknown, we propose that it will lie in a parallelogrammatic region containing the two minima, bounded by $\vec{p}$ and $\vec{q}$, where the minimum energy path is expected to be found. We interpret this observation of requisite movements along both $\vec{p}$ and $\vec{q}$ to mean that PT and ET must be concerted to complete the overall PCET process.

In the ET excited state (Figure 3d), only one minimum is found and it is located above the transition state of the ground state PES. The absence of additional minima in the excited state suggests that the charge‐separated states are not stable for the phenol/phenoxy pair. The small energy gap of 0.11 eV between the ground and excited states at the position of the transition state (see Table 1) indicates a weakly coupled system and CPET proceeding in the nonadiabatic regime.

We are not aware of similarly extensive studies for the indole/indolyl pair as for the phenol/phenoxy pair. For tryptophane/water, CPET has been observed in biological and synthetic settings, noting though that a sequential mechanism is also accessible depending on the strength of the oxidant and pH range [32, 33]. The ground state PES of the indole/indolyl radical pair (Figure 3b) exhibits strikingly similar features to that of the phenol/phenoxy pair. The $\vec{p}$ and $\vec{q}$ vectors have a similar angle to that of the phenol/phenoxy pair, implying that PT and ET are concerted here, too. A notable difference to the phenol/phenoxy system is the smaller separation of the product minimum and transition state from the $\vec{p}$ vector. The system thus needs much less movement along $\vec{q}$ to reach the transition state and the product minimum. This type of quantifiable insight is an advantage of the approach we propose, though further studies involving more examples are needed to make firm connections with experimental data.

The shallow low‐energy region of the excited ET state (Figure 3e) resembles three connected minima. This implies more stable charge‐separated species than in the phenol/phenoxy case. The energy gap at the transition state is again small (see Table 1), implying PCET in the nonadiabatic regime.

### 2.2 Toluene/Benzyl Radical Pair: Hydrogen Atom Transfer

The toluene/benzyl radical pair reacts via a HAT mechanism [24, 25, 28]. If the transferred electron resides in the 1s orbital of the H atom, this should result in a $\vec{p}$ vector directly connecting the reactant and product minima, that is, no distinct movement along the ET vector should be needed to reach the transition state or the product minimum. Gratifyingly, this is precisely what the PES reveals, see Figure 3c. Please note the different scales of the *x*‐ and *y*‐axes compared to Figure 3a,b.

The topology and qualitative energetic characteristics of the PES are strikingly different than for the previous examples: a larger separation of the minima in the $\vec{p}$ direction and a significantly higher energy of the TS and the second minimum are found. The three minima in the excited state surface of the benzyl/toluene pair (Figure 3f) suggest stable charge‐separated species, hence a small coupling between the quantum proton transfer states in the scope of the Hammes–Schiffer model. The large potential coupling ($V_{\mathrm{ab}}$ in Table 1) suggests an adiabatic process as is expected for HAT [27].

## 3 Discussion of the Results

The potential energy surfaces for the three examples above clearly demonstrate the differing behavior of CPET and HAT systems. To rationalize the observations and point out some finer details of the approach we introduce herein, we now discuss the results in more detail.

In any chemical reaction, the movement on a PES from reactant minimum to product minimum is related to changes of nuclear coordinates. Illustrative videos of plausible classical paths for the three examples discussed herein are provided as Supporting Information. With the proton and electron transfer coordinates, $\vec{p}$ and $\vec{q}$, we provide the basis functions for any *mandatory* movement between reactant and product minima. Additional movements will also be thermally accessible. However, we surmise that such movements do not contribute to the reaction, in analogy to our discussion of the ET coordinate obtained from *ab initio* calculations [29].

Inspecting now more closely the CPET systems—the phenol/phenoxy and indole/indolyl radical pairs—one observes that the product minimum cannot be reached by a movement along the PT coordinate $\vec{p}$ exclusively. Purely from the visual inspection of the PESs, we propose that the proton movement, though dominant, is insufficient and that additional nuclear movements along $\vec{q}$ are needed to enable electron transfer and thus complete the overall PCET process.

Quite in contrast, on the PES for the HAT system—the toluene/benzyl radical pair—we observe that movement purely along the PT coordinate $\vec{p}$ suffices to reach the product minimum. In other words, a motion along the ET coordinate $\vec{q}$ does not offer any stabilization or advantage to complete the PCET process. This implies that the motion of the proton directs all relevant changes in the electronic structure of the toluene/benzyl pair, which is consistent with the picture of the electron traveling in the H 1s orbital (see the Supporting Information for the shapes of the donor/acceptor orbitals).

**FIGURE 4:**
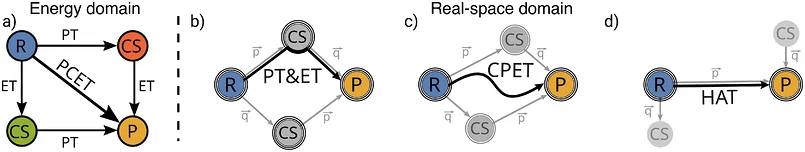
Schematic description of PCET mechanisms in the (a) energy domain and (b–d) real‐space domain based on the results shown in Figure 3, where R denotes reactants, P denotes products, and CS denotes the two different charge‐separated species. Proton transfer ($\vec{p}$) and electron transfer ($\vec{q}$) coordinates are represented by grey arrows, while reaction pathways are indicated by black arrows. (a) Traditional energy based square scheme for reference. (b) Sequential mechanism in which PT and ET are not coupled. (c) Concerted proton–electron transfer (CPET) where nuclear movements along $\vec{p}$ and $\vec{q}$ are needed, resulting in a curved path. (d) Hydrogen atom transfer (HAT), where the reaction proceeds solely along $\vec{p}$. Note that the relative energies of R, P and CS are qualitatively indicated by the number of contour lines around the symbol.

These observations make the reaction path for the HAT system clear immediately: the lowest energy path coincides with $\vec{p}$ and the transition structure lies on this path, see Figure 4d for a schematic depiction. The charge‐separated states lie much higher in energy along $\vec{q}$, on an excited state potential energy surface. Considering the symmetry of the ground state PES with respect to $\vec{p}$, a quantum mechanical path, such as expressed by the center of a nuclear wave packet, should coincide with the classical path. We note that it is justified in this case to only consider the ground state PES because HAT is an adiabatic process, which is reflected in the large energy separation of the relevant states, see Table 1.

For the CPET systems, the situation is more flexible: any low‐energy path in the parallelogram spanned by $\vec{p}$ and $\vec{q}$ is plausible. We propose an S‐shaped reaction path, which initially follows $\vec{p}$, then aligns with $\vec{q}$ to reach the transition structure and finally follows $\vec{p}$ again to reach the product minimum, see Figure 4c for a schematic depiction and representative animations in the Supporting Information. Such a path permits a low‐energy route from reactants to products. Note that this conception of a minimum‐energy path only applies to an adiabatic transfer of classical nuclei. The real reaction path will be strongly influenced by nuclear quantum and nonadiabatic effects. From the former one might expect the system to avoid the transition structure region completely and proceed through regions with narrow barriers, the so‐called “corner cutting” [34, 35]. From the latter, one would expect the reaction to evolve toward the minimum of the excited state. A clearer picture of the reaction paths in CPET will require simulations where these effects are appropriately accounted for; we will return to this point in the next section.

Lastly, we discuss a process for which no specific example is given here, where ET and PT are decoupled. In this scenario, the reaction proceeds through either of the charge‐separated species. As depicted schematically in Figure 4b, the reaction may first proceed along $\vec{p}$ to a charge‐separated state, and then along $\vec{q}$ to the product state (or vice versa). The relative lengths of $\vec{p}$ and $\vec{q}$ will depend on the specific case of interest; however, we expect that $\vec{p}$ and $\vec{q}$ will be orthogonal since the two processes are decoupled.

We now examine the different shapes of the elongated minima in the excited state PESs of the CPET systems, which are indicative of different degrees of stabilization of the charge‐separated species; pronounced minima would indicate stable charge‐separated states, whereas a shallow potential with a single minimum above the transition state indicates destabilized charge‐separated species. Within the Hammes–Schiffer model, this reflects different coupling strengths between quantum proton transfer states: larger for phenol/phenoxy and smaller for indole/indolyl. We note that these features might be difficult to spot in a single, one‐dimensional potential energy curve, whereas they are easy to observe in the approach presented here yielding two‐dimensional PESs.

The results and discussion show that the approach for mapping the energy‐based square scheme into real‐space coordinates offers multiple unique features for interpreting PCET processes: (i) distinct shapes for CPET versus HAT, (ii) access to descriptors for key points on the PES and magnitudes of coupling, (iii) information on the importance of charge‐separated states by inspection of the relevant excited‐state PES. In the following, we will discuss the approach from a conceptual perspective, noting shortcomings and pointing out solutions that can be pursued in the future.

## 4 Discussion of the Approach

In its present form, the approach introduced here uses classical nuclei to discuss how the system will proceed over the PES, which as a bare result is an approximation given the known relevance of nuclear quantum effects for the proton in PCET reactions. This means that as of now, we cannot expect to obtain quantitative rates directly from the PESs. To account for nuclear quantum effects, the vibrational states could be reconstructed in (preferably diabatized) PESs, for example, by using a Fourier grid Hamiltonian [36] or the spectral method [37]. We note that this would allow to not only treat the transferring proton quantum mechanically, but also any other nuclei whose movements are involved in the overall PCET process. Based on our previous work on ET processes [29, 30], we expect that heavy‐atom tunneling may be relevant for the nuclear movements involved in $\vec{q}$ given the narrow barrier widths on the order of 10ths of Å.

Reconstructing the vibrational states is currently hampered by the asymmetry of the PESs (see Figure 3 for the difference in energy of the reactant and product minima). The PESs are asymmetric because they are expressed in terms of electronic energies and are not based on fully relaxed structures except for the reactant minimum. Because our approach only isolates the nuclear movements that are associated with PT and ET, an additional relaxation movement is required to reach the true product minimum (see Supporting Information and additional supporting material for illustrative animations). For instance in the phenol/phenoxy example, the two aromatic rings undergo a reorientation motion, while in the toluene/benzyl system, the acceptor atom has to change hybridization from sp^2^ to sp^3^. To resolve the asymmetry of the PESs and deal with the relaxation movement, there are two readily conceivable solutions: (i) symmetrizing the PESs about the TS region, which may introduce some uncertainty; (ii) employing the nuclear coordinates $\vec{p}$ and $\vec{q}$ as biases in metadynamics, which would be computationally demanding [38]. We note that the proton coordinate may deviate from a simple linear motion, for instance, if large relaxation motions are present that affect the global positioning of the acceptor atom. In such cases, the definition of the proton coordinate would have to reflect this, for example, by using internal coordinates.

Returning to the question of how to properly account for the quantum nature of the proton(s), it is conceivable to obtain the collective nuclear motion for the proton transfer in much the same manner as we do for the electron transfer. The nuclear coordinate for electron transfer is obtained by extracting from an ensemble only those motions that contribute to electron transfer according to the Marcus model [29]. The very same idea can be applied to a proton that is described fully quantum, for example, with the nuclear‐electronic orbital (NEO) method [39]. This would mean that one isolates the collective nuclear movement that leads to a change of the quantum proton state. More specifically, one would first generate an ensemble of distorted geometries, and find the excited state in which the quantum proton is transferred to the acceptor. As the next step, one would fit the normal coordinates of the system to the excitation energies of this state to construct the nuclear coordinate of the proton. This idea cannot be executed at the moment because the calculation of vertical excitation energies for proton transfer states with NEO is currently not established in the literature.

As noted above, our approach cannot describe the full quantum complexity of PCET processes for reasons that are largely rooted in technical restrictions (e.g., asymmetry of the potentials, smoothing of the PESs for calculation of vibronic proton states, calculation of vertical excitation energies for NEO protons). Nevertheless, our approach offers a novel perspective on PCET processes in terms of a PES spanned by two well‐defined real‐space dimensions corresponding to the nuclear movements associated with electron and proton transfer. With the resulting PESs, quantitative comparisons between PCET systems are possible, and we expect that CPET and HAT examples will have clearly distinct shapes.

## 5 Conclusions

Proton‐coupled electron transfer chemistry has been largely discussed in terms of energy‐based square schemes. We herein propose a novel approach which translates the well‐established square scheme from the energy domain to a real‐space domain. This is achieved by employing two well‐defined nuclear coordinates that capture all relevant nuclear movements related to electron and proton transfer. These two independently identified cuts through the (3N−6)‐dimensional space thus span a two‐dimensional surface that incorporates all nuclear motions that are relevant to the overall PCET process. We emphasize that the approach is straightforward to implement using only routine quantum chemistry tools.

For well‐known test cases representing concerted proton and electron transfer (phenol/phenoxy and indole/indolyl radical pairs) and hydrogen atom transfer (toluene/benzyl radical pair), we observed clearly distinct features in the PESs: (i) qualitative differences in the spatial separation of minima for CPET and HAT, (ii) qualitative differences in the energetics of CPET and HAT, (iii) clear characteristics of CPET versus HAT in terms of the $\vec{p}$ and $\vec{q}$ orientations with respect to the reactant and product minima, (iv) straightforward identification of the relevance of charge‐separated states from the shape of the excited state PESs. We believe that the results presented herein can contribute to a better distinction of PCET scenarios by paving the way to clear‐cut definitions, for example, for coupled or concerted electron–proton transfer and HAT. Studying a larger number of examples will provide insights into trends, for example, regarding the variance in the relative orientation of the nuclear coordinates associated with ET and PT or the variability of the key descriptors of the PESs listed above.

## Conflicts of Interest

The authors declare no conflicts of interest.

## Supporting information




**Supporting File 1**: anie73657‐sup‐0001‐SuppMat.pdf.

**Supporting File 2**: anie73657‐sup‐0002‐SuppVideos.zip.

## Data Availability

The data that support the findings of this study are available in the Supporting Information of this article. This includes: computational details; identification of ET states; orthogonalization procedure; discussion of the CASSSCF/NEVPT2 scans; 1D cuts through the PESs; relaxation motions; optimized structures; ET and proton coordinates. Additional references are cited in the Supporting Information [25, 26, 28, 29, 31, 40, 41, 42, 43, 44, 45, 46, 47, 48, 49, 50, 51, 52, 53, 54]. Animations of molecular structures traversing the calculated potential energy surfaces are shared alongside this article. Input files, structures and potential energy surfaces as raw data are available at: doi.org/10.5281/zenodo.20204569.
