# Supplementary material for: Mapping Proton‐Coupled Electron Transfer With Real Space Coordinates
Source: Angew Chem Int Ed Engl. 2026 Jul 23;65(39):e5050035. doi: 10.1002/anie.5050035 (PMC13592303; doi:10.1002/anie.5050035)
Supplement: Supplementary file 1 [file ANIE-65-e5050035-s001.pdf]

Supporting Information: Mapping Proton-Coupled Electron Transfer with Real Space Coordinates

Adam Šrut,† Martin Diefenbach,† Marvin L. Kronenberger,† Benjamin J. Lear,*,‡ and Vera Krewald*,†

†TU Darmstadt, Department of Chemistry, Quantum Chemistry, Peter-Grünberg-Straße 4, 64287 Darmstadt, Germany

‡The Pennsylvania State University, Department of Chemistry, University Park, PA 16802, USA

E-mail: bul14@psu.edu; vera.krewald@tu-darmstadt.de

Contents

1 Computational details **S-3**

2 Identification of the ET states **S-6**

3 TD-DFT results **S-7**

4 Orthogonalization procedure **S-8**

5 Discontinuities in 2-dimensional scans **S-10**

6 1D cuts through calculated surfaces **S-12**

7 Active space composition in 1D scans **S-13**

8 ET state for toluene/benzyl pair **S-15**

9 Relaxation motions **S-17**

10 Comparison with TD-DFT **S-19**

11 Proof-of-concept identification of ET dimensions in PCET systems **S-21**

12 Optimized structures **S-22**

13 PCET coordinates **S-25**

References **S-37**

1 Computational details

The calculations needed for thermal sampling and identifying the ET coordinate were performed with density functional theory using the local hybrid density functional[S1–S3] LH20t[S4] with the def2-TZVP basis set[S5] as implemented in the TURBOMOLE package.[S6] The resolution of identity approximation for computation of the Coulomb integrals[S7] was used. The convergence criterion for the self-consistent field method was set to $10^{-8}\,E_\mathrm{h}$. The D4 dispersion correction[S8] was used. The excited states were determined using TD-DFT without the Tamm–Dancoff approximation.

The methodology for obtaining the ET coordinates is described in detail in our previously published work.[S9] In short, the phase space of all studied molecules was sampled using the Wigner sampling method as implemented in the SHARC package[S10] at a temperature of 300 K. 500 structures were generated as representative of the ensemble. The ET coordinate was then obtained as a linear combination of normal modes, where the expansion coefficients are determined by fitting the normal coordinates to the excitation energy of the ET state. Even though the studied systems exhibit a strong multi-reference character in the transition state region, the DFT-based sampling will still deliver a meaningful results since it is confined to the Franck–Condon region, where the multi-reference character is not expected.

The inner reorganization energies (λ^in) for ET were calculated using the Huang–Rhys factors. These are defined as projections of normal modes (q_i) to the ET coordinate (q_ET): $S_i = |\langle \vec{q}_i | \vec{q}_\mathrm{ET} \rangle|$. The reorganization energy is the sum of the Huang–Rhys factors weighted by the corresponding vibrational frequencies: $\lambda = \sum_i S_i \hbar\omega_i$.

The proton coordinate was defined as a linear motion of the proton from its equilibrium position towards the acceptor atom (O for phenol/phenoxy, N for indole/indolyl and C for toluene/benzyl). All other atoms were frozen in the configuration of the optimized minimum. Prior studies use the same approach but the atoms of donor and acceptor were arranged at the configuration of the transition state.[S11–S13] Herein, we use the configuration of the optimized geometry because the ET coordinate is defined using the normal modes at the optimized

equilibrium structure. We note that other definitions of a proton coordinate are conceivable, such as based on the donor-H stretching mode or using different linear interpolation or fitting schemes.

The potential energy landscapes were constructed from CASSCF-based wavefunctions using the nuclear coordinates spanned by ET and PT motions. Computations were performed with the ORCA program package[S14,S15] employing state-averaged CASSCF calculations followed by multireference perturbational treatment using quasi-degenerate NEVPT2.[S16–S18] The CASSCF wavefunctions were constructed from an active space which comprises three electrons distributed over the two highest occupied and the two lowest unoccupied π orbitals, giving rise to a CAS(3,4) expansion. The orbitals were optimised by the average over the lowest seven doublet states, employing the def2-TZVP basis set[S5] along with the RIJK algorithm for fitting of the Coulomb and exchange integrals in conjunction with the def2/JK auxiliary basis sets.[S19] Final energies were obtained from NEVPT2 calculations to account for dynamical correlation effects.

The overall computational strategy is based on the definition of two relevant coordinates and subsequent calculation of a two-dimensional potential energy surface spanned by those coordinates. Here we provide a step-by-step description of the methodology:

Step 1: The electron transfer coordinate is defined using our approach outlined in Ref. S9. The coordinate space of the system is sampled using the Wigner sampling method. For each geometry in the ensemble, the electronic structure is solved and excited states are calculated. Geometries are then converted to normal coordinates. Then a linear fit of the normal coordinates to the excitation energy of the ET state is performed. The coefficients of the fit are used to construct the ET coordinate as a linear combination of normal modes.

Step 2: The proton coordinate is defined as a linear motion of the proton from its optimized position towards the acceptor atom. Formally, this coordinate has the same form as a normal mode (*i.e.* a vector of linear displacements from an equilibrium geometry).

Step 3: The coordinates are orthogonalized by forming their sum and difference. Note

that the newly formed coordinates must be renormalized by appropriate normalization factors. Scalar products of these coordinates with the original coordinates are calculated to determine the orientations of the ET vector $\vec{q}$ and PT vector $\vec{p}$ on the potential energy surface calculated in the next step.

Step 4: Potential energy surfaces spanned by sum and difference coordinates of the ground state and excited ET state are reconstructed. The electronic structure is solved using the CASSCF/NEVPT2(def2-TZVP) level of theory. The reference structure that also defines the origin of the coordinates is the optimized structure of the reactants.

2 Identification of the ET states

In order to perform a multi-component fit to the excitation energy in the thermally populated ensemble,[S9] the ET state needs to be identified. To that end we use the natural transition orbitals (NTOs) obtained from TD-DFT calculations at the optimized geometry (LH20t/def2-TZVP level of theory), see Figure S1. For the phenol/phenoxy and indole/indolyl pairs, the ET state was found to be the first excited state. For the toluene/benzyl pair, the ET state is fifth excited state at the optimized geometry. The fitting was then performed to the excitation energies of these states in the ensembles. Energies and oscillator strengths of all calculated excited states are listed in Tables S1 and S2, respectively.

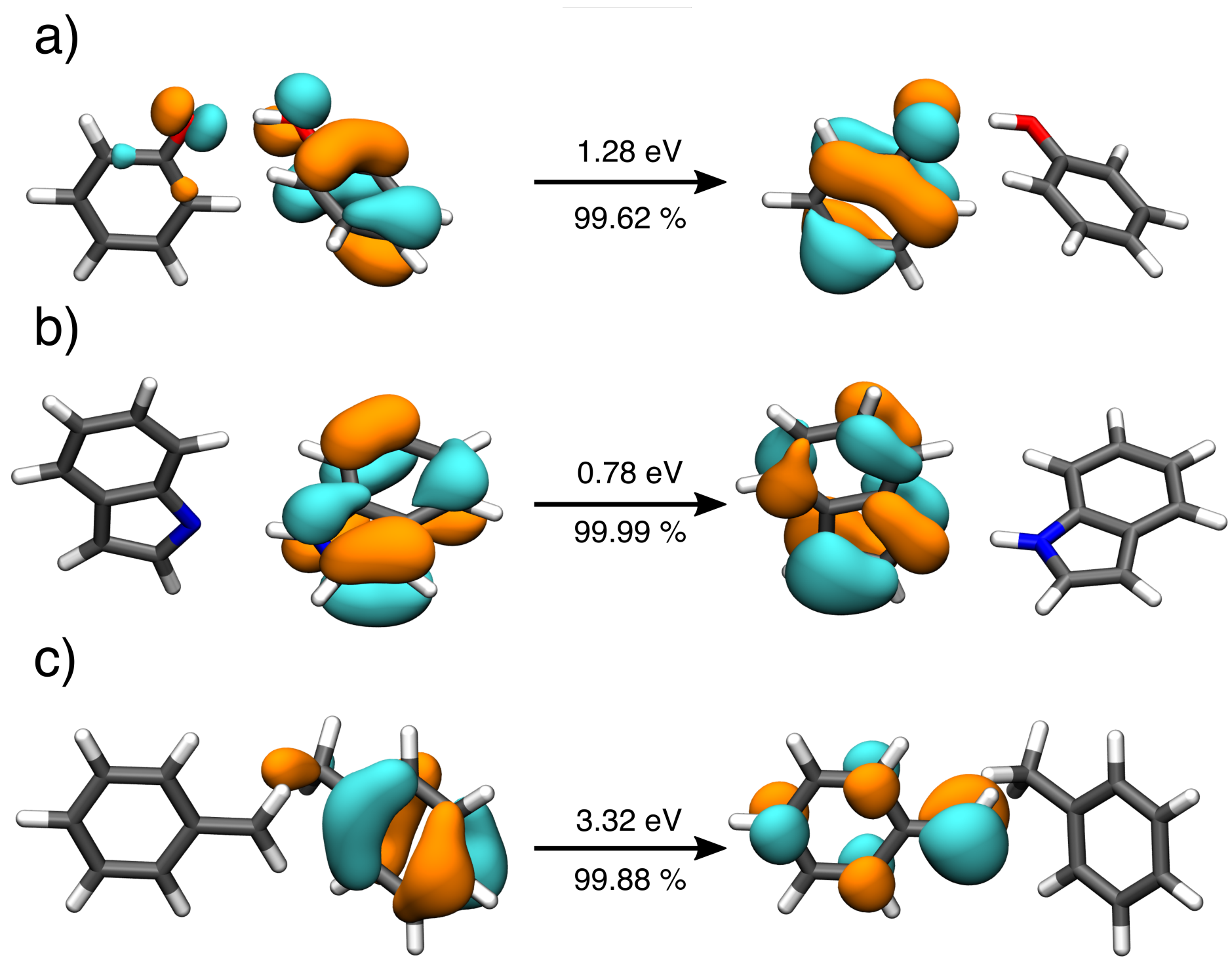

Figure S1: a) NTOs of the ET state for phenol/phenoxy pair. b) NTOs of the ET state for indole/indolyl pair. c) NTOs of the ET state for the toluene/benzyl pair.

3 TD-DFT results

Energies and oscillator strengths of all calculated excited states are listed in Tables S1 and S2, respectively. The TD-DFT calculations were done at LH20t/def2-TZVP level of theory utilizing the optimized geometries.

Table S1: Energies (in eV) of excited states at optimized geometries.

Excited state	Phenol/Phenoxy	Indole/Indolyl	Toluene/Benzyl
1	1.27970	0.78780	3.32671
2	1.40442	1.23525	3.51085
3	2.25964	1.24973	3.97297
4	2.33055	2.40394	4.14172
5	3.64884	2.68801	4.19939
6	4.09754	2.92069	4.49437
7	4.29868	3.53990	4.68250
8	4.38029	3.94026	4.71244

Table S2: Oscillator strengths of excited states at optimized geometries.

Excited state	Phenol/Phenoxy	Indole/Indolyl	Toluene/Benzyl
1	0.00042	0.00003	0.00014
2	0.00012	0.00002	0.00275
3	0.00550	0.00004	0.03002
4	0.00115	0.00017	0.00001
5	0.04503	0.04625	0.00072
6	0.00000	0.00001	0.00004
7	0.00003	0.00000	0.00075
8	0.00012	0.00922	0.00073

4 Orthogonalization procedure

To translate the non-orthogonal electron transfer coordinate ($\vec{q}$) and the proton coordinate ($\vec{p}$) into a set of orthogonal axes, we take inspiration from molecular orbital theory. If we want to study an interaction of electrons in two atomic orbitals (AOs), one possibility is to view the problem directly in the basis of these orbitals. Alternatively, we can form two molecular orbitals (MOs) as positive and negative linear combinations of the AOs. One MO will be the sum of the AOs (bonding MO), the other the difference of the AOs (antibonding MO). In contrast to the AOs, the MOs will now be orthogonal to each other, i.e. they will have a zero overlap.

We used a sum and a difference of the electron transfer ($\vec{q}$) and the proton transfer ($\vec{p}$) coordinates to construct the two-dimensional scans presented in the main text. Below, we provide a mathematical description of our methodology.

We start with two normalized vectors $\vec{p}$ and $\vec{q}$ describing the proton and electron transfercoordinates, respectively. These are $3N$-vectors of linear displacements from a reference structure (optimized geometry). Their scalar product measures an overlap between those two coordinates:

$$S = \vec{q} \cdot \vec{p} = \sum_{i=1}^{3N} q_i p_i \tag{1}$$

The new coordinate system is constructed as a sum ($\vec{s}$) and a difference ($\vec{d}$) of proton and eletron transfer coordinates:

$$\vec{s} = \frac{1}{\sqrt{2(1+S)}} (\vec{q} + \vec{p}) \tag{2a}$$

$$\vec{d} = \frac{1}{\sqrt{2(1-S)}} (\vec{q} - \vec{p}) \tag{2b}$$

We can now easily show that $\vec{s}$ and $\vec{d}$ are mutually orthogonal:

$$\vec{s}\cdot\vec{d} = \left[\frac{1}{\sqrt{2(1+S)}}\left(\vec{q}+\vec{p}\right)\right]\cdot\left[\frac{1}{\sqrt{2(1-S)}}\left(\vec{q}-\vec{p}\right)\right] \tag{3a}$$

$$= \frac{1}{2\sqrt{1-S^2}}\left(\vec{q}\cdot\vec{q}-\vec{p}\cdot\vec{p}-\vec{q}\cdot\vec{p}+\vec{p}\cdot\vec{q}\right) \tag{3b}$$

$$= \frac{1}{2\sqrt{1-S^2}}\left(1-1-S+S\right) \tag{3c}$$

$$= 0 \tag{3d}$$

and also normalized:

$$\vec{s}\cdot\vec{s} = \left[\frac{1}{\sqrt{2(1+S)}}\left(\vec{q}+\vec{p}\right)\right]\cdot\left[\frac{1}{\sqrt{2(1+S)}}\left(\vec{q}+\vec{p}\right)\right] \tag{4a}$$

$$= \frac{1}{2(1+S)}\left(\vec{q}\cdot\vec{q}+2\vec{q}\cdot\vec{p}+\vec{p}\cdot\vec{p}\right) \tag{4b}$$

$$= \frac{1}{2(1+S)}\left(1+2S+1\right) \tag{4c}$$

$$= 1 \tag{4d}$$

$$\vec{d}\cdot\vec{d} = \left[\frac{1}{\sqrt{2(1-S)}}\left(\vec{q}-\vec{p}\right)\right]\cdot\left[\frac{1}{\sqrt{2(1-S)}}\left(\vec{q}-\vec{p}\right)\right] \tag{5a}$$

$$= \frac{1}{2(1-S)}\left(\vec{q}\cdot\vec{q}-2\vec{q}\cdot\vec{p}+\vec{p}\cdot\vec{p}\right) \tag{5b}$$

$$= \frac{1}{2(1-S)}\left(1-2S+1\right) \tag{5c}$$

$$= 1 \tag{5d}$$

5 Discontinuities in 2-dimensional scans

The slight discontinuities in the phenol/phenoxy and indol/indolyl PESs are due to reordering of the frontier MO eigenvalues upon moving to higher-energy regions. Figures S2 and S3 show selected examples for two coordinate pairs with rotation of two frontier MOs.

5.1 Phenol/Phenoxy

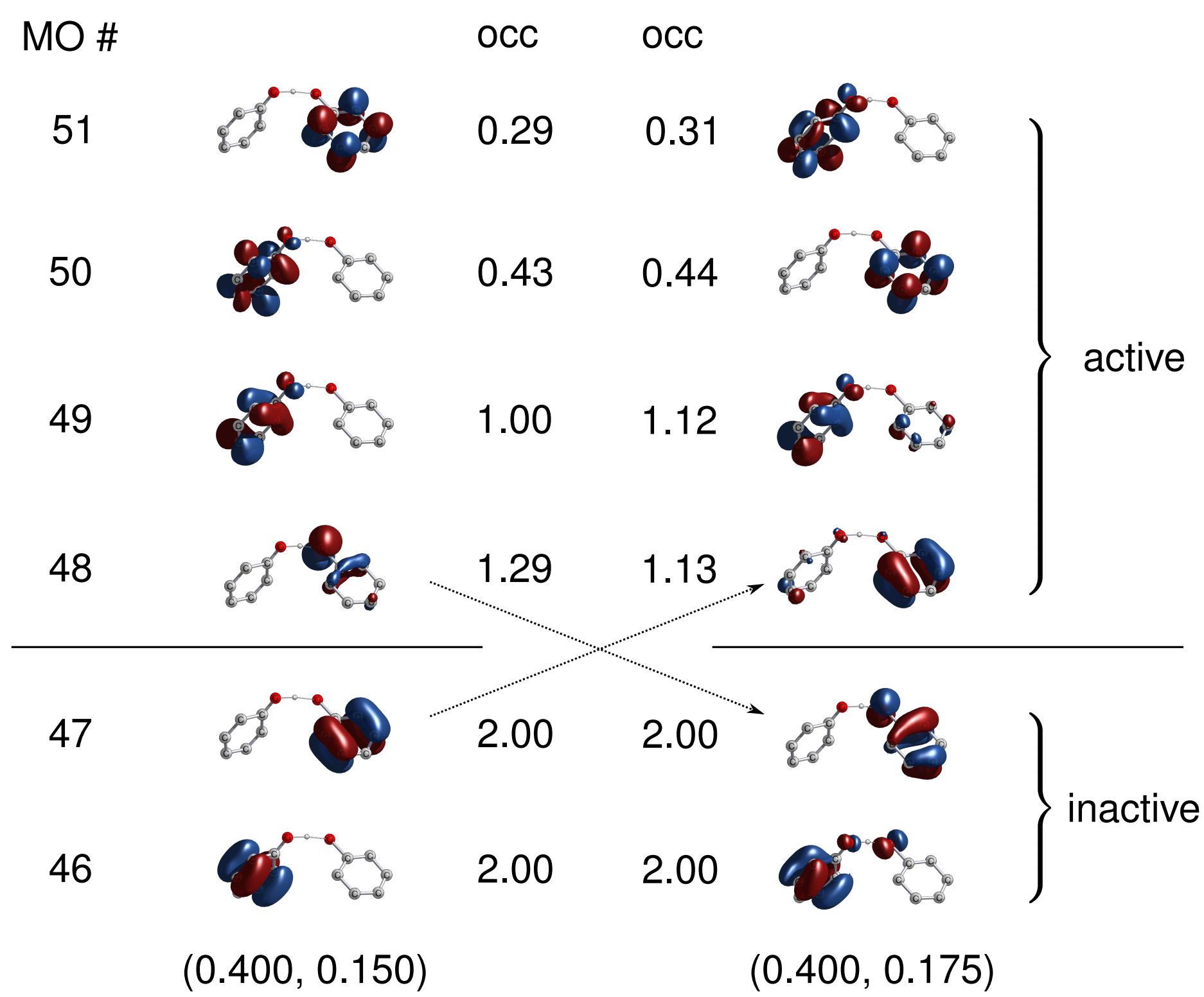

Figure S2: Rotation of active and inactive frontier molecular orbitals in the region of discontinuities, shown for coordinate pairs (0.400, 0.150) and (0.400, 0.175) of the potential energy surface of the phenol/phenoxy system.

5.2 Indole/Indolyl

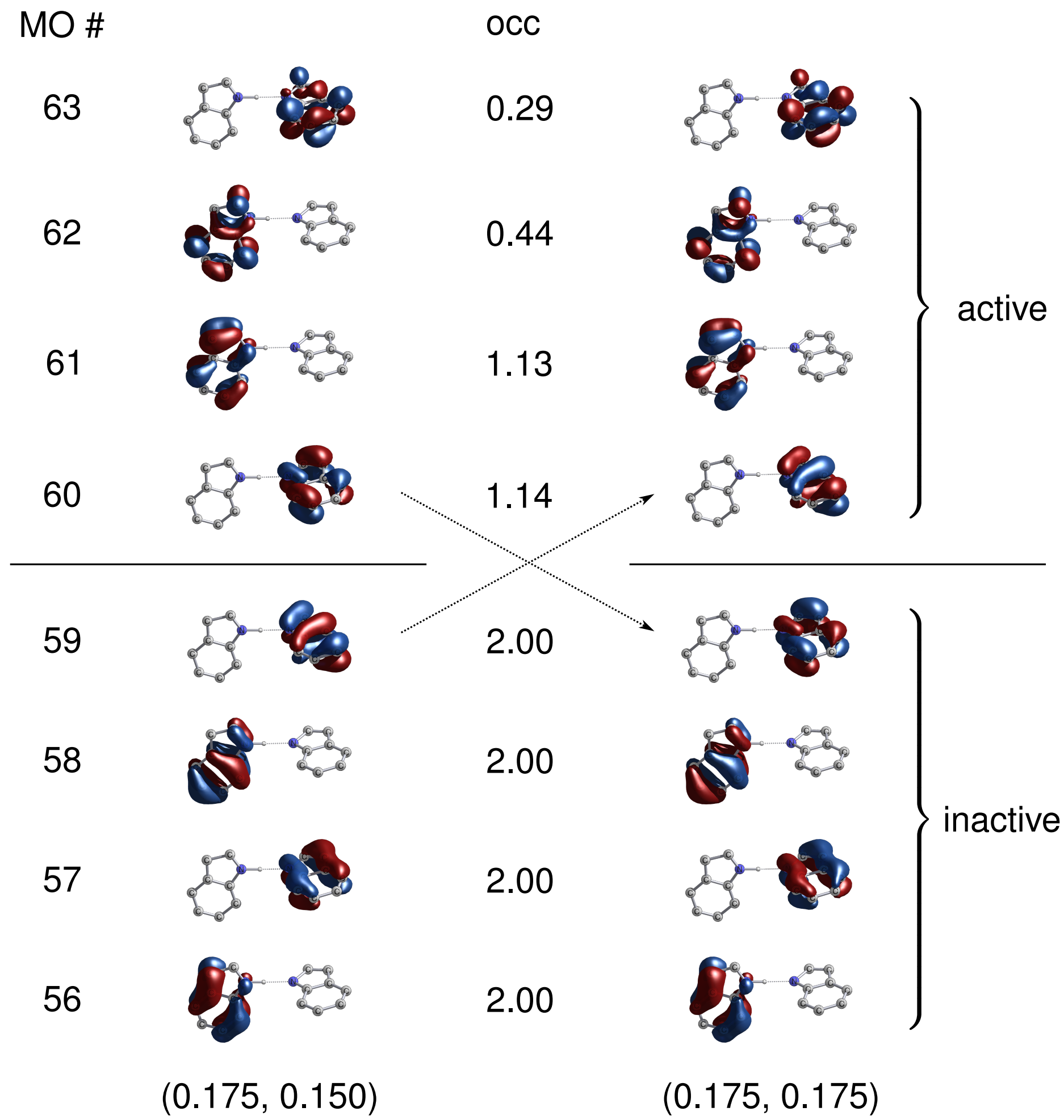

Figure S3: Rotation of active and inactive frontier molecular orbitals in the region of discontinuities, shown for coordinate pairs (0.175, 0.150) and (0.175, 0.175) of the potential energy surface of the indol/indolyl system. The number of nodal planes and the natural occupation numbers of MO 60 are unchanged.

6 1D cuts through calculated surfaces

In the main text, we mention that in the case of HAT mechanism the transferring electron should be confined to the H 1s orbital. Here, we show a progression of the electronic structure changes along the linearly interpolated coordinates between the identified minima of the 2D scan. The changes in the donor and acceptor orbitals clearly indicate a significant participation of the H 1s orbital. A detailed discussion of the excited state characters is given in Section 8.

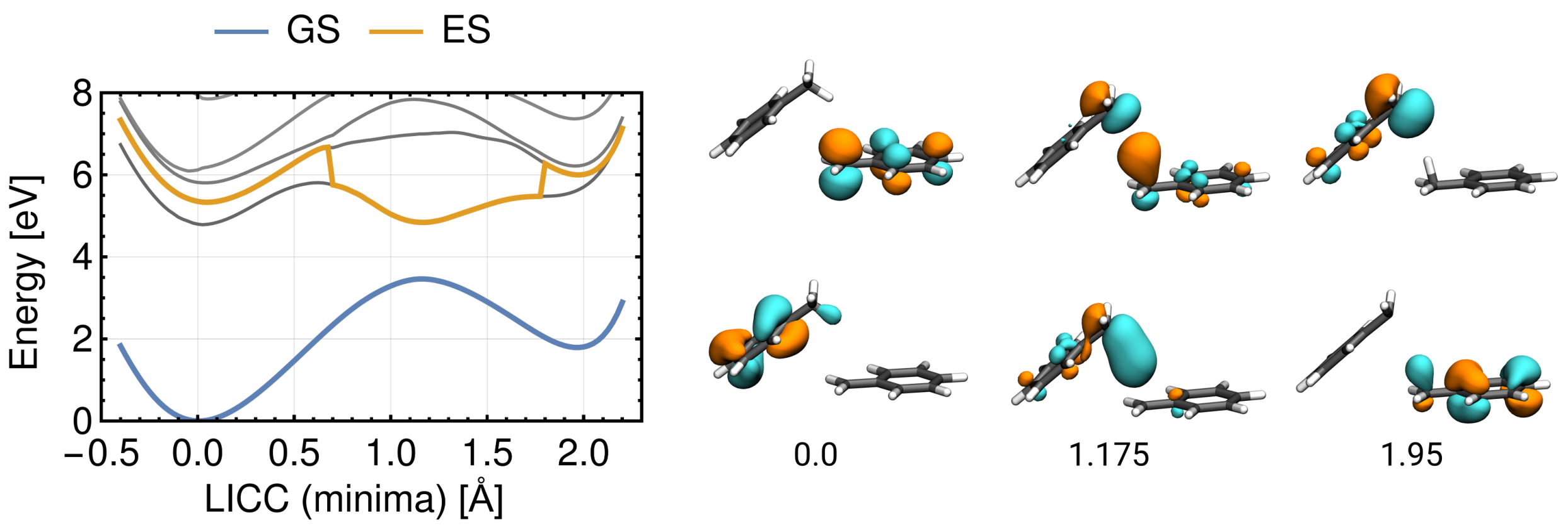

Figure S4: Potential energy curves along linear interpolation between the minima of the 2D scan (left).

7 Active space composition in 1D scans

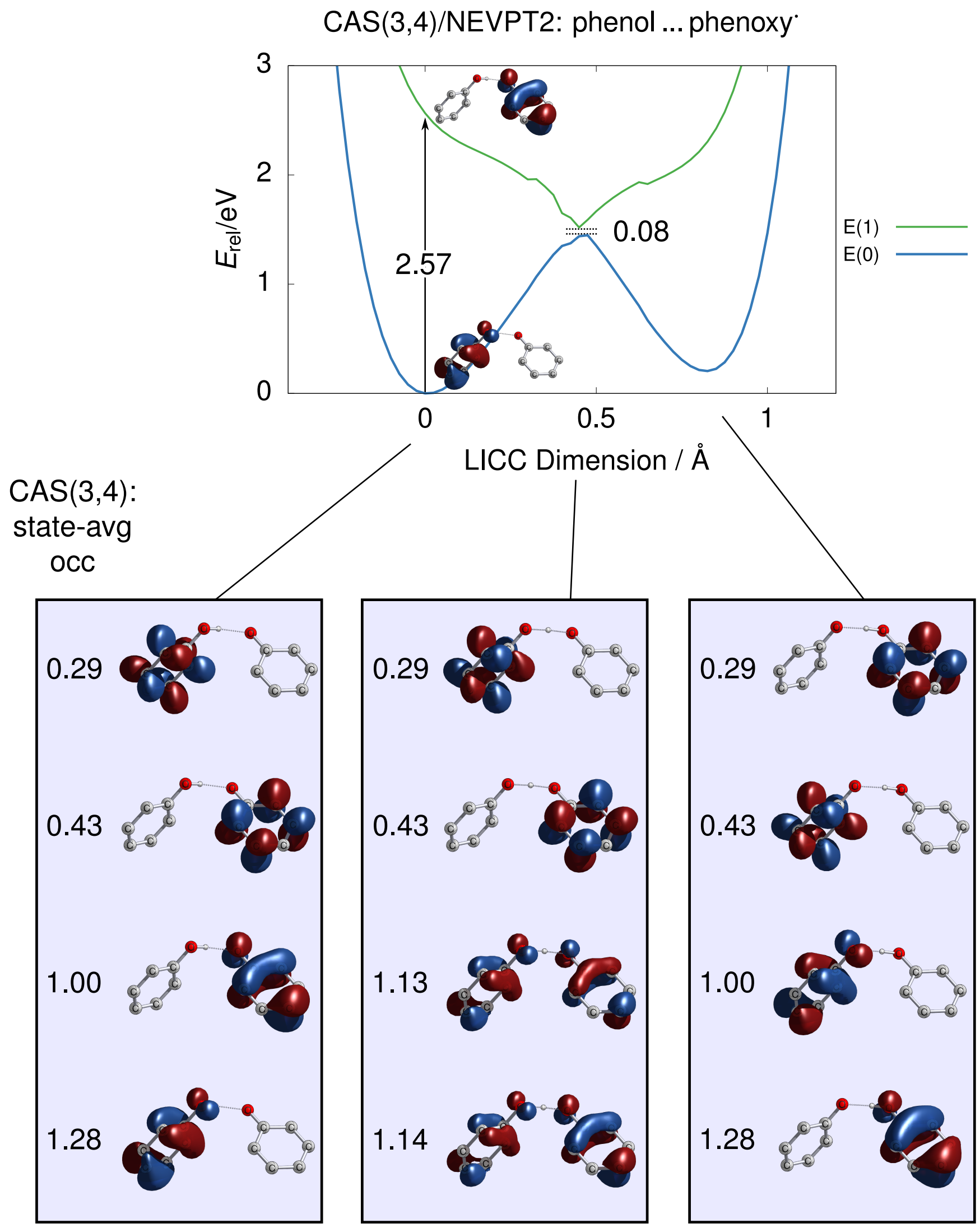

Figure S5: Active space composition in the scan connecting the two potential minima of the 2D dimensional scan for the phenol/phenoxy system.

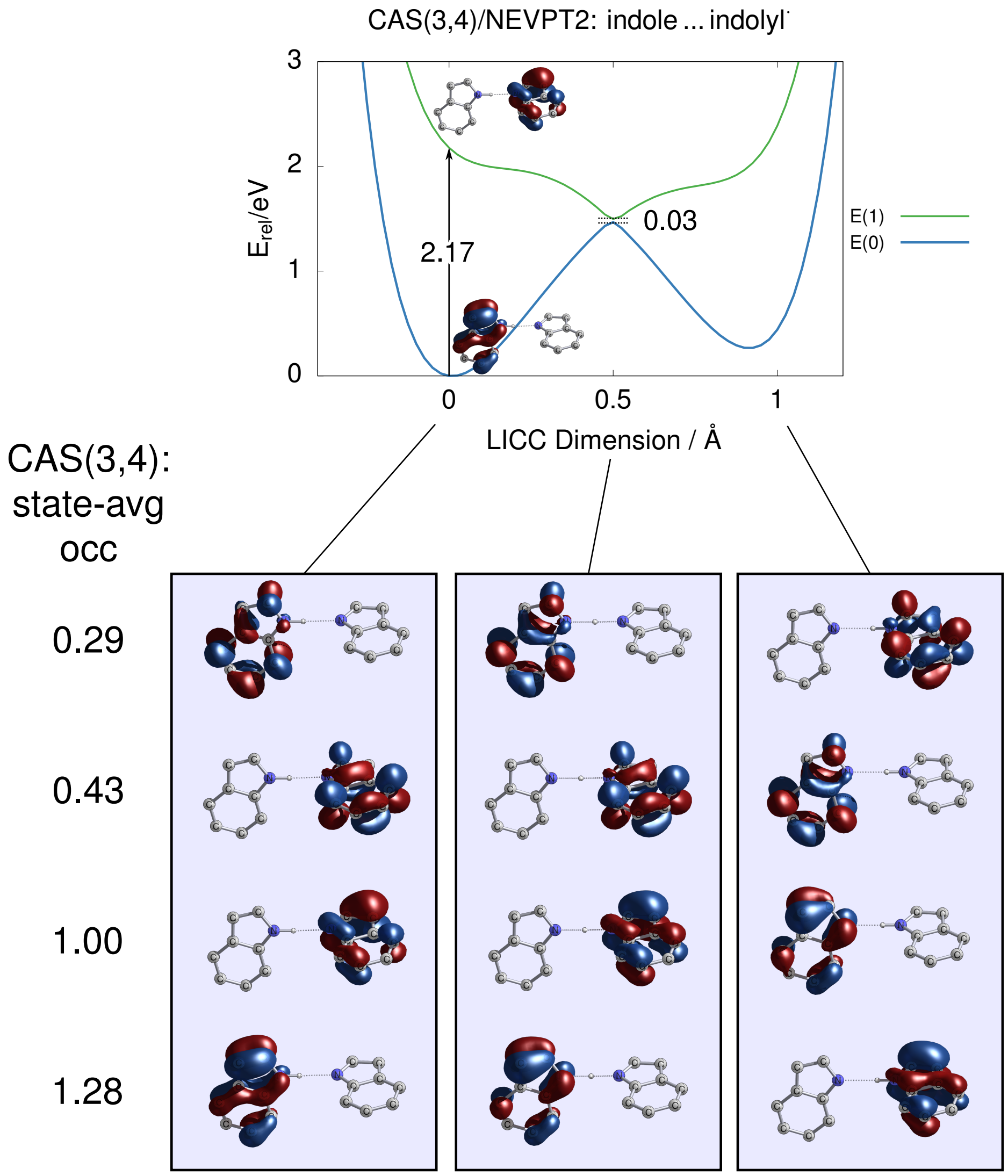

Figure S6: Active space composition in the scan connecting the two potential minima of the 2D dimensional scan for the indol/indolyl system.

8 ET state for toluene/benzyl pair

The identification of the ET state for the toluene/benzyl pair is a challenging task in comparison to the phenol/phenoxy and indol/indolyl systems. At similar energies as the ET state there are other excited state without ET character. In the one- and two-dimensional scans the ET state needs to be disentangled from the other states. This is done by checking the leading configuration in the CASSCF wavefunction: if the leading configuration corresponds to the ET event (in comparison to the leading configuration of the ground state) then the electronic state is assigned as the ET state. In Figure S7, a discontinuity in the potential of the ET state is apparent. This is caused by a multi-configurational character of the first two excited states.

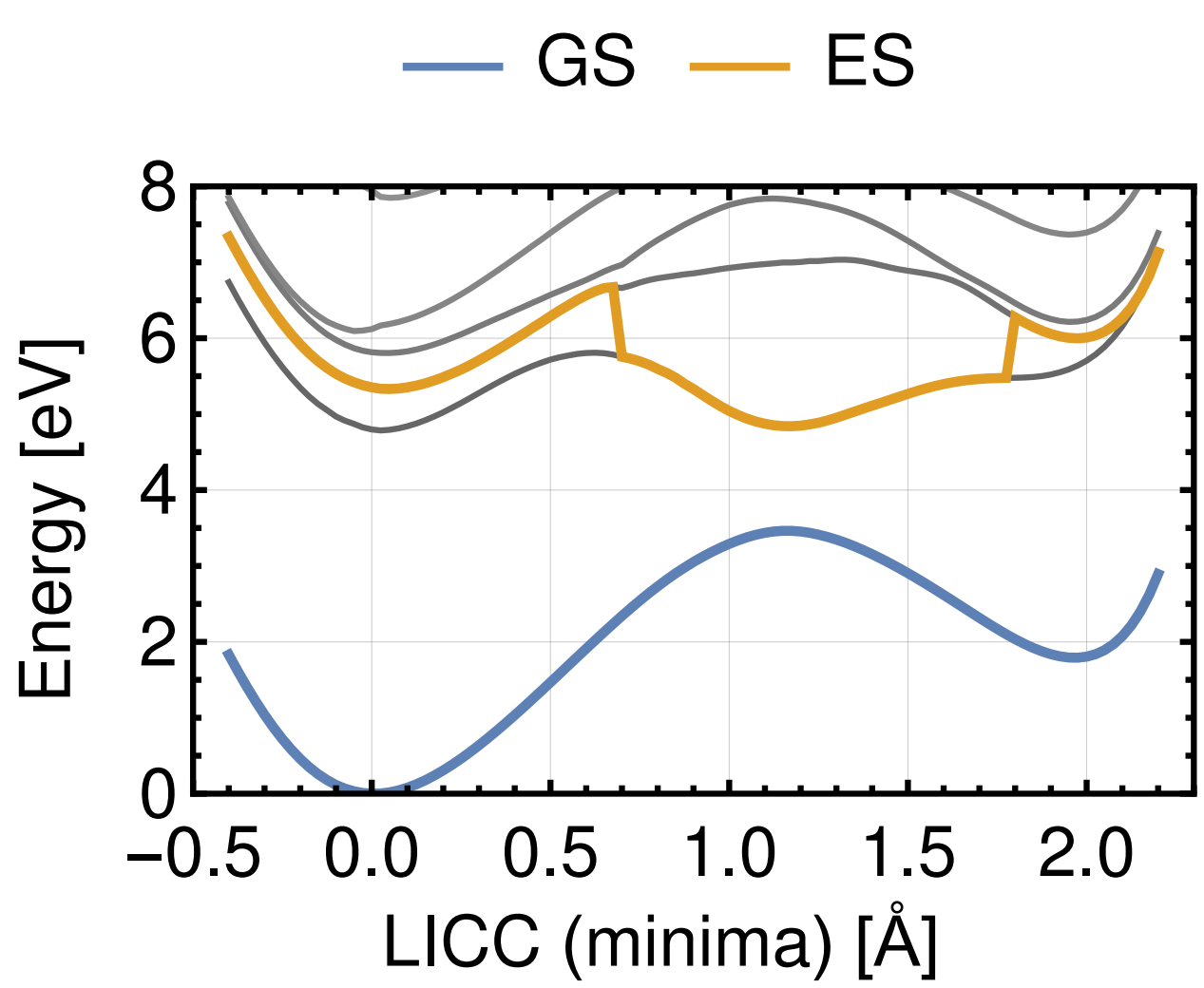

Figure S7: One-dimensional cut through the PESs. The x-axis was obtained by linear interpolation in the Cartesian coordinates (LICC) between the minima of the 2D scan. The blue trace depicts the ground state, the orange trace depicts the electron transfer state. The calculations are performed at the CASSCF(3,4)/NEVPT2 level of theory.

In Figure S8 and Table S3 we present the active spaces and compositions of the first four electronic states for geometries 0.675 and 0.700 Å, where the discontinuity in the ET state appears. The active spaces are almost identical and thus the configuration state functions are comparable. The ground state composition is in both cases led by configuration 2100 (using the active space ordering from Figure S8), the corresponding ET configuration is 1200.

Note, that the first two excited states are multi-configurational and they both contain an appreciable amount of ET character. It is thus difficult to decide which state should be labeled as the ET state.

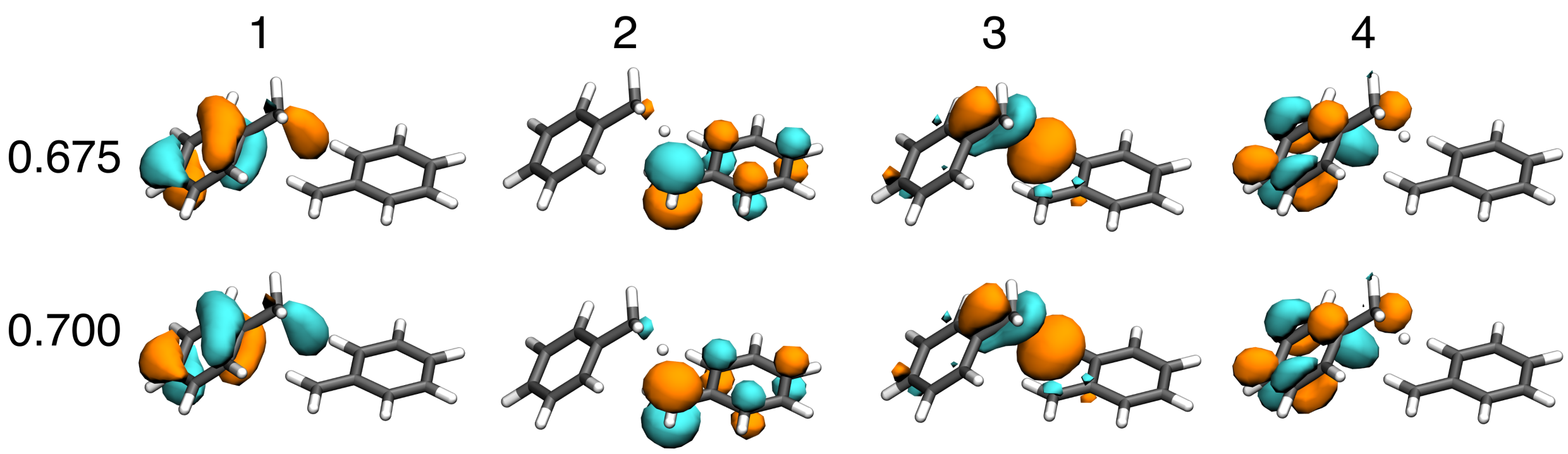

Figure S8: Active space at positions 0.675 and 0.700 Å of the one-dimensional scan presented in Figure S7.

Table S3: Weights of the leading configurations in the wavefunctions for two points in the scan when the discontinuity in the ET state occurs. Configurations are given in occupation number of active orbitals based on ordering presented in Figure S8.

Geometry	Configuration	Ground state	Ex. state 1	Ex. state 2	Ex. state 3
0.675	2100	0.956	0.005	0.005	0.000
	1200	0.024	0.236	0.349	0.017
	1110	0.003	0.507	0.137	0.038
	1101	0.000	0.125	0.308	0.204
0.700	2100	0.953	0.006	0.003	0.000
	1200	0.027	0.259	0.256	0.075
	1110	0.003	0.503	0.151	0.036
	1101	0.000	0.086	0.151	0.377

We find the reason for the multi-configurational character in using the optimized geometry as a reference (i.e. not having a relaxed geometries along the scanned pathways). The H atom is thus transferred from a carbon in tetrahedral configuration to a carbon in planar configuration. Allowing for a smooth transfer between these configurations would probably remove the multi-reference character of the excited states. This is, however, beyond the scope of this work.

9 Relaxation motions

The presented scans of the potential energy surfaces showed an offset of the product minimum to higher energies. This is because we choose our reference geometry (point [0,0] in all 2D scans) to be the optimized structure. In contrast, other studies work with a symmetrized system.[S11,S13,S20] We, however, define our ET coordinate from the normal modes at the optimized minimum. Using a transition state structure as a reference would spoil the ET coordinate. In turn, we observe a shift of the product minimum towards higher energies. Below, we demonstrate that the nuclear motions needed to reach the product minimum are of little importance for the PCET processes.

In Figure S9 we present motions that will reach the global product minimum. These were obtained by linear interpolation in Cartesian coordinates from the product minimum of the 2D scan to the optimized geometry. All three motions in Figure S9 describe mostly relative reorientations of the aromatic rings. For the toluene/benzyl pair this motion will also result in a planarization of the $-CH_2^{\cdot}$ group and the rearrangement of the newly formed $-CH_3$ group to tetrahedral geometry. The indole/indolyl pair exhibits a large motion confined to C−N and C−C stretches; these become only apparent because the net motion of the rings is small. The differences between the relaxation motions and the ET and PT coordinates are quantified in Table S4.

Table S4: Overlaps of the relaxation motions ($\vec{\Delta}$) with proton transfer ($\vec{p}$) and electron transfer ($\vec{q}$) coordinates.

	Phenol/Phenolate	Indol/Indolyl	Toluene/Benzyl
$\vec{\Delta} \cdot \vec{q}$	0.041	-0.170	0.044
$\vec{\Delta} \cdot \vec{p}$	0.082	-0.193	0.201

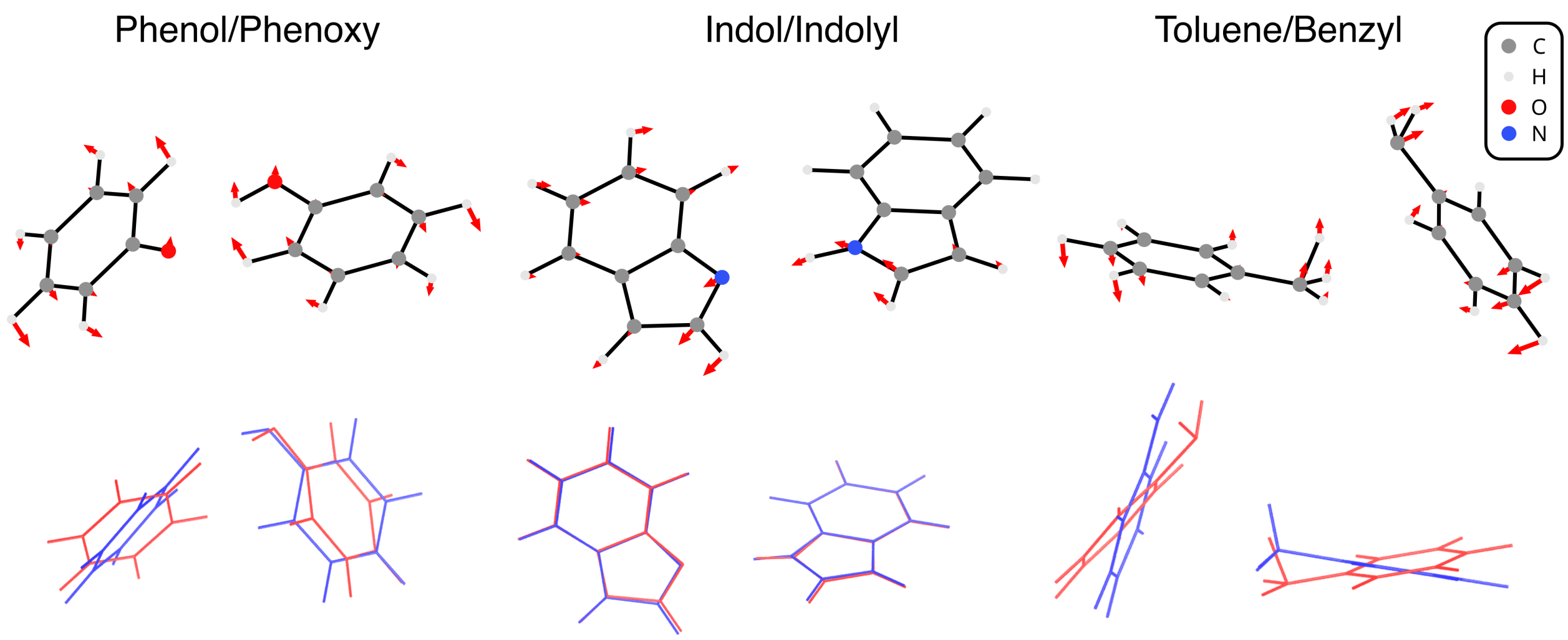

Figure S9: Top: Relaxation motions that bring the system to the true product minimum. The motions are obtained by linear interpolation in Cartesian coordinates from the product minimum in 2D scan to the optimized structure. Bottom: Optimized structures (blue) and structures of the product minima of 2D scans (red). Structure optimization done at LH20t/def2-TZVP level of theory.

10 Comparison with TD-DFT

10.1 Phenol/Phenoxy pair

Comparing the two-dimensional scans calculated at the CASSCF/NEVPT2 level of theory with DFT calculations (employing the LH20t functional) shows only minor differences in the ground state, see Figure S10a-b. However, the potential energy surfaces of the ET excited state are qualitatively different, Figure S10c-d. The potential energy surface calculated with TD-DFT has two minima placed above the ground state minima, while the surface calculated using CASSCF/NEVPT2 has only one minimum located above the transition state region. Furthermore, the TD-TDFT excited state surface shows a set of discontinuities: these are situated along the intersection seam of the two electronic states. This is not surprising, since DFT should not provide reliable results in regions with degenerate or nearly degenerate electronic states.

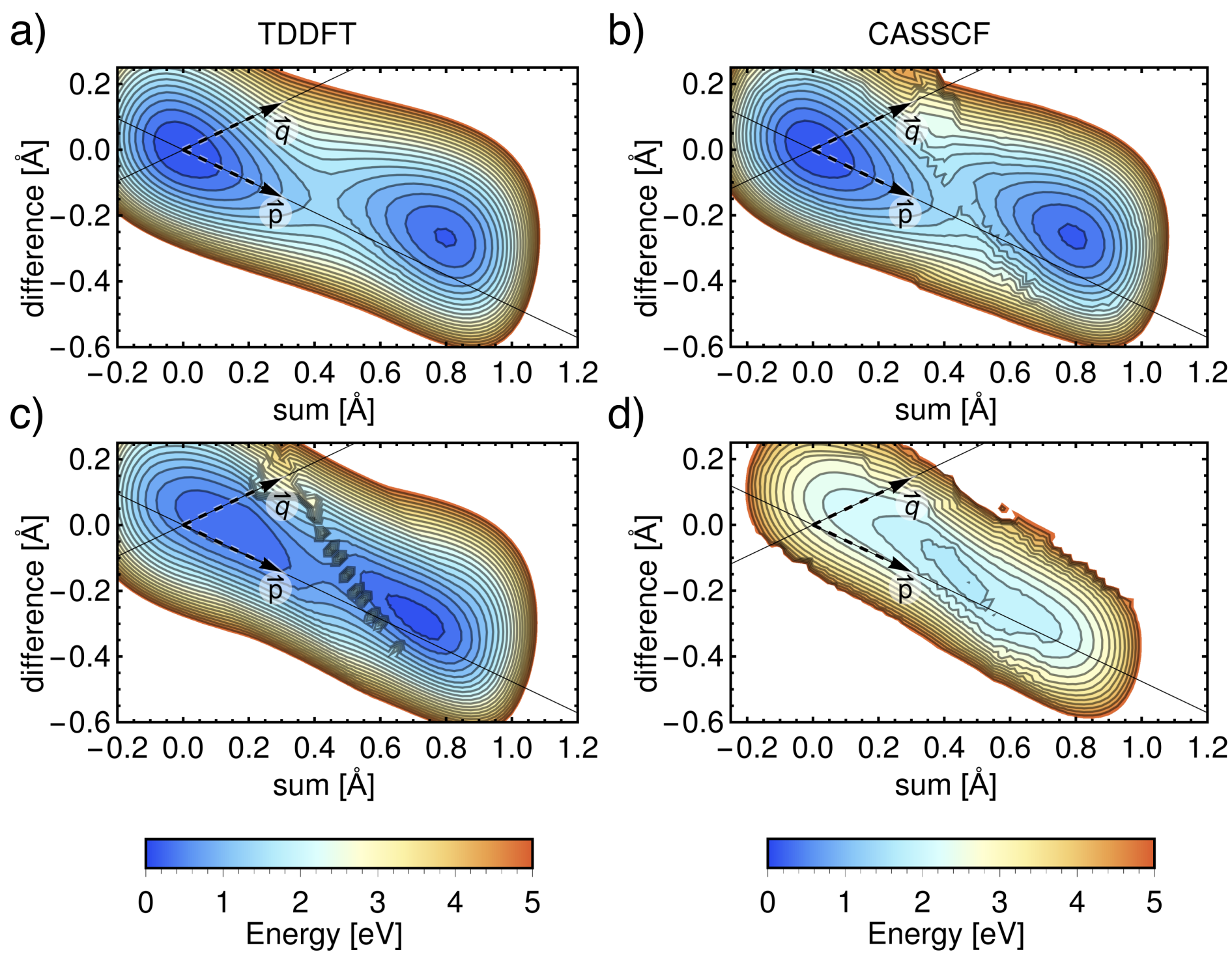

Figure S10: a-b) Ground state potential energy surfaces calculated at the DFT and CASSCF/NEVPT2 levels of theory, respectively. c-d) Excited state potential energy surfaces calculated at the TD-DFT and CASSCF/NEVPT2 levels of theory, respectively.

10.2 Indole/Indolyl pair

The comparison of the two-dimensional scans calculated at the CASSCF/NEVPT2 level of theory and with DFT (employing the LH20t functional) is very similar to the phenol/phenoxy pair presented above. Figure S11a-b shows only minor differences on the ground state PES. However, there are the same qualitative differences in the excited state PES, see Figure S11c-d.

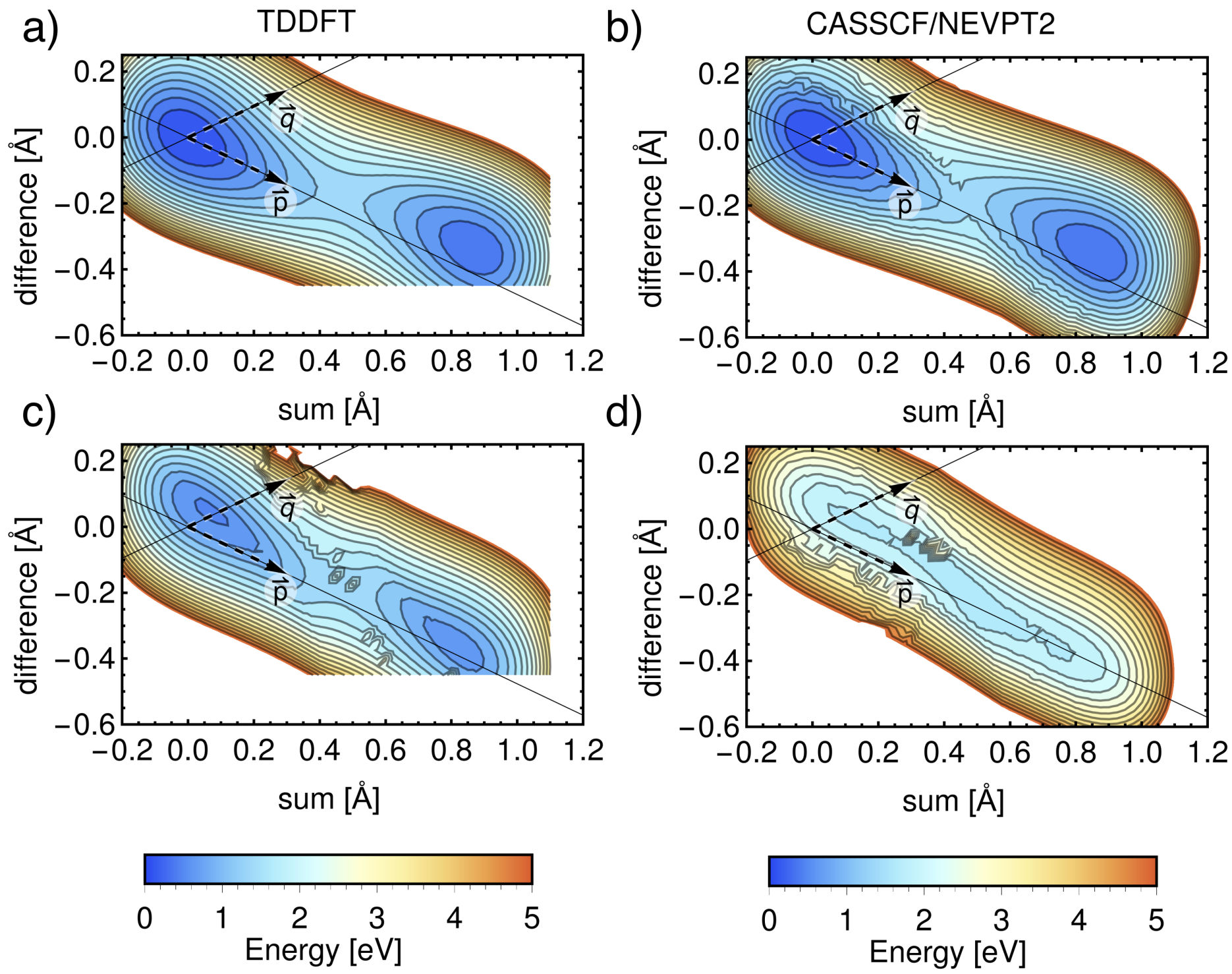

Figure S11: a-b) Ground state potential energy surfaces calculated at the DFT and CASSCF/NEVPT2 levels of theory, respectively. c-d) Excited state potential energy surfaces calculated at the TD-DFT and CASSCF/NEVPT2 levels of theory, respectively.

11 Proof-of-concept identification of ET dimensions in PCET systems

Our methodology for identifying the ET coordinate of the Marcus–Hush model, presented in Ref. S9, was first applied to asymmetric PCET system of phenol/amine and phenoxy/ammonia pairs. The identified ET coordinates are depicted in Figure S12. In both cases, the motion of the transferring proton is involved in the ET coordinate, much more prominently for phenol/amine pair. Another noteworthy part of these coordinates is the elongation/contraction of the C–O bond. This leads to a stabilization/destabilization of the orbital with the unpaired electron at the phenol moiety. Such a motion will thus have a large impact on the ET process.

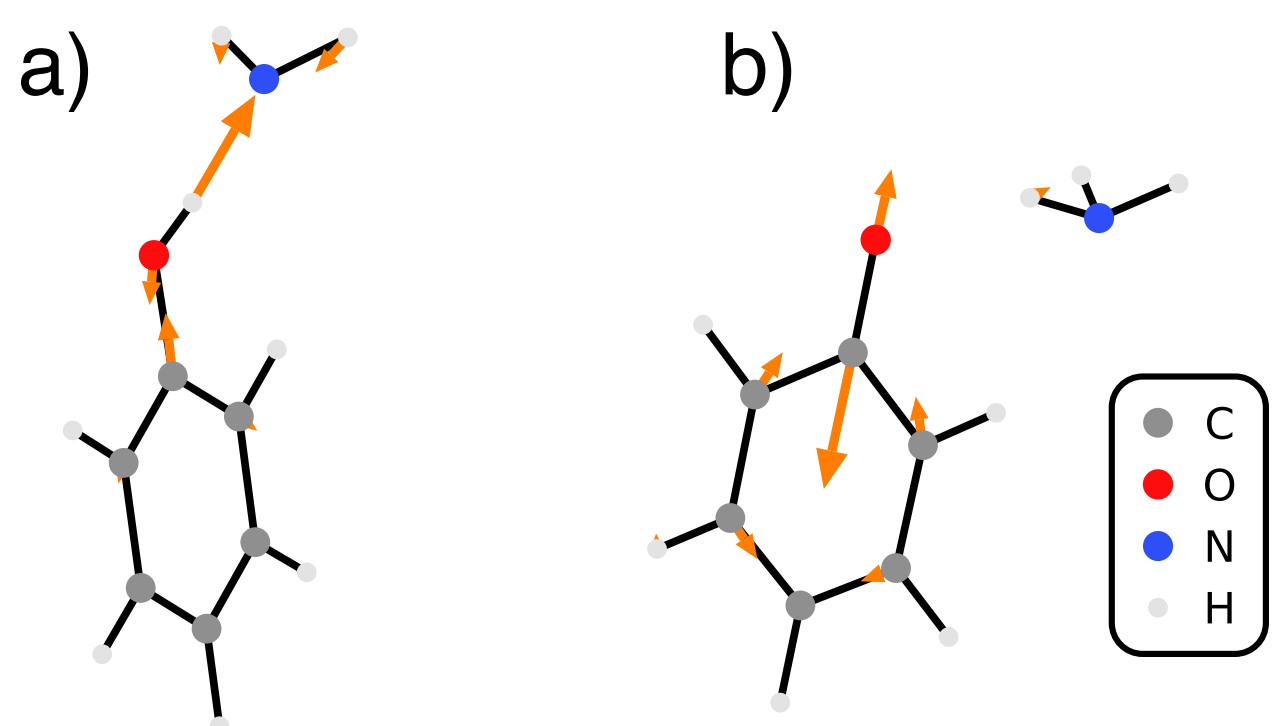

Figure S12: a) ET coordinate for the phenol/amine pair. b) ET coordinate for the phenoxy/ammonia pair.

12 Optimized structures

Below, the optimized structures of all species are listed. These structures correspond to point [0,0] in the two-dimensional scans. Optimizations were done with DFT using the LH20t functional and the def2-TZVP basis set.

Phenol/Phenoxy pair

```
25
total energy (lh20t/def2-TZVP) = -613.50497324732 Eh
   C    -1.95472     0.93993     0.25701
   C    -2.95498     0.73880     1.20526
   C    -3.88640    -0.26965     1.02511
   C    -3.83575    -1.09136    -0.09514
   C    -2.83784    -0.88734    -1.03667
   C    -1.89768     0.12117    -0.86937
   H    -2.98435     1.38150     2.07176
   H    -4.65874    -0.41681     1.76586
   H    -2.78666    -1.51272    -1.91606
   H    -1.12899     0.28599    -1.61161
   H    -4.56354    -1.87612    -0.23307
   O    -1.06719     1.93250     0.48613
   H    -0.35601     1.93602    -0.17908
   O     1.25990     1.74460    -1.02487
   C     2.03341     0.87186    -0.56816
   C     3.43543     0.88352    -0.91005
   C     4.27720    -0.07673    -0.42037
   C     3.78154    -1.08666     0.41833
   C     2.42589    -1.12606     0.77530
   C     1.56091    -0.17378     0.30806
   H     3.77753     1.67706    -1.55684
   H     5.32606    -0.06417    -0.67583
   H     2.06877    -1.91310     1.42208
   H     0.51228    -0.17518     0.56816
   H     4.45392    -1.84326     0.79406
```

Indole/Indolyl pair

```
31
total energy (lh20t/def2-TZVP) = -725.98866094143
   C    -0.62752     1.28508    -2.43310
   C    -0.61891     1.85426    -3.69127
   C    -0.60708     1.06048    -4.85018
   C    -0.60346    -0.31684    -4.77086
   C    -0.61197    -0.92361    -3.50995
   C    -0.62424    -0.10621    -2.35577
   H    -0.63766     1.89481    -1.54139
   H    -0.62186     2.92994    -3.78806
   H    -0.60136     1.54189    -5.81701
   H    -0.59481    -0.91836    -5.66849
   C    -0.61350    -2.27792    -3.05303
   C    -0.62640    -2.23134    -1.68995
   H    -0.60626    -3.16691    -3.65796
   H    -0.63244    -3.03517    -0.97459
   N    -0.63127    -0.93034    -1.26233
   H    -0.65486    -0.63140    -0.28931
   C     1.77403     0.16434     2.01963
   C     2.74000     0.41610     3.01019
   C     2.38777     0.59733     4.34186
   C     1.05468     0.53609     4.73990
   C     0.08573     0.28805     3.76787
   C     0.46267     0.10237     2.41290
   H     2.05631     0.02414     0.98687
   H     3.78033     0.46940     2.72649
   H     3.15726     0.78813     5.07407
   H     0.78344     0.67790     5.77579
   C    -1.32082     0.15991     3.78089
   C    -1.69485    -0.09863     2.42075
   H    -1.98517     0.23655     4.62551
   H    -2.69942    -0.25356     2.05840
   N    -0.66836    -0.13649     1.61212
```

Toluene/Benzyl pair

```
29
total energy (lh20t/def2-TZVP) = -541.65211810005 Eh
   C     2.90387     0.55618    -0.35813
   C     1.95849     1.02709     0.58862
   C     2.13514     2.34387     1.08528
   C     3.18546     3.12854     0.66280
   C     4.10295     2.64076    -0.26783
   C     3.95100     1.34951    -0.77288
   C     0.89525     0.22349     1.01579
   C    -2.21077     0.42649    -1.45963
   C    -2.41672    -0.84723    -0.67895
   C    -3.14503    -0.85300     0.50759
   C    -3.29122    -2.01624     1.25115
   C    -2.71126    -3.19950     0.81630
   C    -1.99015    -3.20935    -0.36975
   C    -1.84761    -2.04358    -1.10924
   H    -3.60291     0.06370     0.85381
   H    -3.85983    -1.99730     2.16953
   H    -2.82645    -4.10586     1.39187
   H    -1.54081    -4.12649    -0.72195
   H    -1.28701    -2.06222    -2.03386
   H    -1.26998     0.89308    -1.16792
   H    -2.16776     0.22609    -2.52743
   H     0.17963     0.58705     1.73645
   H     1.42801     2.72599     1.80776
   H     3.29957     4.12881     1.05450
   H     4.92559     3.25858    -0.59485
   H     4.66032     0.96856    -1.49314
   H     2.79175    -0.44497    -0.74972
   H    -3.01305     1.13477    -1.27119
   H     0.76354    -0.77686     0.63501
```

13 PCET coordinates

We provide the nuclear coordinates as linear atomic displacements from a reference geometry (listed in Section 12).

13.1 Electron transfer dimension

Phenol/Phenoxy pair

```
25
ET dimensions; displacements from reference geometry in Angstrom
   C   0.2169390   0.1560309  -0.0228950
   C  -0.1157065  -0.0302362   0.0997548
   C   0.0452785   0.0874690   0.0631531
   C  -0.0161790   0.0142940  -0.0095862
   C   0.1206606   0.0701343  -0.0194091
   C  -0.0389982  -0.1052678  -0.1250463
   H   0.0049636   0.0303025   0.0354839
   H  -0.0036090   0.0324906   0.0338736
   H   0.0055225  -0.0408359  -0.0479971
   H   0.0543342  -0.0075212  -0.0081274
   H   0.0634089   0.0092736   0.0257462
   O  -0.1917800  -0.1273861   0.0158569
   H   0.5504938   0.0254336  -0.3210511
   O  -0.2370017   0.1898571  -0.0814918
   C   0.2593284  -0.2536449   0.1318287
   C  -0.1430725   0.0492380  -0.0169179
   C  -0.0160085  -0.1524313   0.1098051
   C  -0.0027944  -0.0151560   0.0067444
   C   0.1657118  -0.0259262  -0.0029270
   C   0.0378142   0.1119364  -0.0960700
   H  -0.0278586   0.0160653  -0.0195323
   H   0.0074927   0.0019798  -0.0186608
   H  -0.0225351   0.0217962  -0.0322917
   H   0.0328790   0.0005210  -0.0060056
   H   0.0284743   0.0335610  -0.0108934
```

Indole/Indolyl pair

```
31
ET dimensions; displacements from reference geometry in Angstrom
   C  -0.0127426  -0.0813118   0.1155033
   C   0.0031414   0.0115308  -0.1134525
   C   0.0109155   0.0355617   0.0437274
   C  -0.0141315  -0.0441002   0.0004754
   C  -0.0154085  -0.0104308   0.0024516
   C   0.0085758   0.0396259  -0.0225488
   H   0.0082589  -0.0094427   0.0284960
   H  -0.0007271  -0.0135815   0.0100859
   H  -0.0133391  -0.0049125  -0.0204328
   H   0.0070937   0.0011652   0.0040176
   C   0.0107571   0.0756816  -0.1973558
   C  -0.0032066   0.1723367   0.2504979
   H   0.0033091   0.0214862   0.0490313
   H  -0.0175338   0.0143365   0.0359252
   N  -0.0019296  -0.1860487  -0.0983423
   H   0.0008855   0.1126229   0.6215606
   C   0.1336057   0.0191278   0.0812839
   C  -0.0043718  -0.0193049  -0.1361505
   C  -0.0999363  -0.0182889   0.0426740
   C   0.0736122   0.0213836   0.0070284
   C  -0.0903631   0.0031414  -0.0016872
   C  -0.2016716  -0.0322432  -0.0713471
   H   0.0179813   0.0090419   0.0012864
   H  -0.0031693   0.0213370   0.0333711
   H   0.0446409   0.0431215   0.0468035
   H  -0.0204142  -0.0048472   0.0034676
   C  -0.0642627  -0.0219709  -0.1378190
   C  -0.0535802   0.0496465   0.3486165
   H   0.0362142  -0.0086411   0.0041294
   H  -0.0072428   0.0183448   0.0084546
   N   0.2715180  -0.0001958  -0.1428154
```

Toluene/Benzyl pair

```
29
ET dimensions; displacements from reference geometry in Angstrom
   C  -0.0696689  -0.1022660   0.0115326
   C  -0.3321416  -0.3104536   0.0890313
   C  -0.0664419   0.0651202   0.0619275
   C   0.1094717   0.0553869  -0.0432536
   C   0.1136909   0.1286895  -0.0315436
   C   0.1455866   0.0654623  -0.0619651
   C   0.0609328   0.0744979  -0.0584747
   C  -0.0460402  -0.1033342   0.0360377
   C   0.0816067   0.3713823  -0.1942515
   C  -0.1782982  -0.0719922   0.2450313
   C  -0.0503757   0.2143562   0.0453262
   C   0.0157650  -0.1655431   0.0552308
   C   0.0529147  -0.0063047  -0.1431314
   C   0.1468803  -0.2108160  -0.0654820
   H  -0.0015670   0.0545446   0.0431376
   H   0.0408916   0.0807329  -0.0500183
   H   0.0426650   0.0719661  -0.0537455
   H   0.0210549   0.0247435   0.0531696
   H  -0.1087694   0.0017908   0.1025616
   H   0.0463763  -0.1421485   0.1732983
   H   0.0399527  -0.1733276   0.0496660
   H   0.0362741   0.0415662   0.0693532
   H   0.0101497  -0.0381172   0.0790270
   H  -0.0142685   0.0869400   0.0021701
   H   0.0422190  -0.0126905   0.0067308
   H   0.0193685   0.0454188  -0.0013462
   H  -0.0610282  -0.0595324   0.0432915
   H  -0.0062203   0.0079376   0.1252598
   H   0.0849646  -0.0396992   0.0007366
```

13.2 Proton coordinate

Phenol/Phenoxy pair

```
25
Proton coordinate; displacements from reference geometry in Angstrom
   C    0.0000000    0.0000000    0.0000000
   C    0.0000000    0.0000000    0.0000000
   C    0.0000000    0.0000000    0.0000000
   C    0.0000000    0.0000000    0.0000000
   C    0.0000000    0.0000000    0.0000000
   C    0.0000000    0.0000000    0.0000000
   H    0.0000000    0.0000000    0.0000000
   H    0.0000000    0.0000000    0.0000000
   H    0.0000000    0.0000000    0.0000000
   H    0.0000000    0.0000000    0.0000000
   H    0.0000000    0.0000000    0.0000000
   O    0.0000000    0.0000000    0.0000000
   H    0.8811350   -0.1043730   -0.4612020
   O    0.0000000    0.0000000    0.0000000
   C    0.0000000    0.0000000    0.0000000
   C    0.0000000    0.0000000    0.0000000
   C    0.0000000    0.0000000    0.0000000
   C    0.0000000    0.0000000    0.0000000
   C    0.0000000    0.0000000    0.0000000
   C    0.0000000    0.0000000    0.0000000
   H    0.0000000    0.0000000    0.0000000
   H    0.0000000    0.0000000    0.0000000
   H    0.0000000    0.0000000    0.0000000
   H    0.0000000    0.0000000    0.0000000
   H    0.0000000    0.0000000    0.0000000
```

Indole/Indolyl pair

```
31
Proton coordinate; displacements from reference geometry in Angstrom
   C   0.0000000   0.0000000   0.0000000
   C   0.0000000   0.0000000   0.0000000
   C   0.0000000   0.0000000   0.0000000
   C   0.0000000   0.0000000   0.0000000
   C   0.0000000   0.0000000   0.0000000
   C   0.0000000   0.0000000   0.0000000
   H   0.0000000   0.0000000   0.0000000
   H   0.0000000   0.0000000   0.0000000
   H   0.0000000   0.0000000   0.0000000
   H   0.0000000   0.0000000   0.0000000
   C   0.0000000   0.0000000   0.0000000
   C   0.0000000   0.0000000   0.0000000
   H   0.0000000   0.0000000   0.0000000
   H   0.0000000   0.0000000   0.0000000
   N   0.0000000   0.0000000   0.0000000
   H  -0.0068683   0.2518840   0.9677331
   C   0.0000000   0.0000000   0.0000000
   C   0.0000000   0.0000000   0.0000000
   C   0.0000000   0.0000000   0.0000000
   C   0.0000000   0.0000000   0.0000000
   C   0.0000000   0.0000000   0.0000000
   C   0.0000000   0.0000000   0.0000000
   H   0.0000000   0.0000000   0.0000000
   H   0.0000000   0.0000000   0.0000000
   H   0.0000000   0.0000000   0.0000000
   H   0.0000000   0.0000000   0.0000000
   C   0.0000000   0.0000000   0.0000000
   C   0.0000000   0.0000000   0.0000000
   H   0.0000000   0.0000000   0.0000000
   H   0.0000000   0.0000000   0.0000000
   N   0.0000000   0.0000000   0.0000000
```

Toluene/Benzyl pair

```
29
Proton coordinate; displacements from reference geometry in Angstrom
   C   0.0000000   0.0000000   0.0000000
   C   0.0000000   0.0000000   0.0000000
   C   0.0000000   0.0000000   0.0000000
   C   0.0000000   0.0000000   0.0000000
   C   0.0000000   0.0000000   0.0000000
   C   0.0000000   0.0000000   0.0000000
   C   0.0000000   0.0000000   0.0000000
   C   0.0000000   0.0000000   0.0000000
   C   0.0000000   0.0000000   0.0000000
   C   0.0000000   0.0000000   0.0000000
   C   0.0000000   0.0000000   0.0000000
   C   0.0000000   0.0000000   0.0000000
   C   0.0000000   0.0000000   0.0000000
   C   0.0000000   0.0000000   0.0000000
   H   0.0000000   0.0000000   0.0000000
   H   0.0000000   0.0000000   0.0000000
   H   0.0000000   0.0000000   0.0000000
   H   0.0000000   0.0000000   0.0000000
   H   0.0000000   0.0000000   0.0000000
   H   0.6879745  -0.2127569   0.6938484
   H   0.0000000   0.0000000   0.0000000
   H   0.0000000   0.0000000   0.0000000
   H   0.0000000   0.0000000   0.0000000
   H   0.0000000   0.0000000   0.0000000
   H   0.0000000   0.0000000   0.0000000
   H   0.0000000   0.0000000   0.0000000
   H   0.0000000   0.0000000   0.0000000
   H   0.0000000   0.0000000   0.0000000
   H   0.0000000   0.0000000   0.0000000
```

13.3 Sum coordinate

Phenol/Phenoxy pair

```
25
Sum coordinate; displacements from reference geometry in Angstrom
   C   0.1201339   0.0864049  -0.0126785
   C  -0.0640746  -0.0167438   0.0552410
   C   0.0250738   0.0484375   0.0349722
   C  -0.0089594   0.0079156  -0.0053085
   C   0.0668180   0.0388382  -0.0107482
   C  -0.0215960  -0.0582940  -0.0692467
   H   0.0027487   0.0167806   0.0196499
   H  -0.0019986   0.0179923   0.0187581
   H   0.0030582  -0.0226136  -0.0265793
   H   0.0300886  -0.0041650  -0.0045007
   H   0.0351138   0.0051354   0.0142574
   O  -0.1062017  -0.0705423   0.0087811
   H   0.7927906  -0.0437141  -0.4331869
   O  -0.1312440   0.1051368  -0.0451276
   C   0.1436078  -0.1404605   0.0730025
   C  -0.0792290   0.0272664  -0.0093686
   C  -0.0088650  -0.0844116   0.0608066
   C  -0.0015474  -0.0083929   0.0037348
   C   0.0917659  -0.0143571  -0.0016209
   C   0.0209403   0.0619868  -0.0532005
   H  -0.0154272   0.0088965  -0.0108164
   H   0.0041492   0.0010963  -0.0103338
   H  -0.0124792   0.0120700  -0.0178821
   H   0.0182074   0.0002885  -0.0033257
   H   0.0157682   0.0185850  -0.0060324
```

Indole/Indolyl pair

```
31
Sum coordinate; displacements from reference geometry in Angstrom
   C  -0.0070577  -0.0450363   0.0639740
   C   0.0017399   0.0063866  -0.0628381
   C   0.0060458   0.0196966   0.0242193
   C  -0.0078270  -0.0244259   0.0002633
   C  -0.0085343  -0.0057773   0.0013579
   C   0.0047499   0.0219476  -0.0124891
   H   0.0045744  -0.0052301   0.0157831
   H  -0.0004027  -0.0075224   0.0055863
   H  -0.0073882  -0.0027209  -0.0113172
   H   0.0039290   0.0006454   0.0022252
   C   0.0059580   0.0419179  -0.1093097
   C  -0.0017761   0.0954524   0.1387436
   H   0.0018328   0.0119006   0.0271571
   H  -0.0097115   0.0079406   0.0198980
   N  -0.0010687  -0.1030471  -0.0544690
   H  -0.0033137   0.2018900   0.8802644
   C   0.0740004   0.0105943   0.0450208
   C  -0.0024214  -0.0106924  -0.0754099
   C  -0.0553519  -0.0101297   0.0236359
   C   0.0407717   0.0118438   0.0038929
   C  -0.0500495   0.0017399  -0.0009345
   C  -0.1117002  -0.0178586  -0.0395171
   H   0.0099593   0.0050080   0.0007125
   H  -0.0017554   0.0118180   0.0184833
   H   0.0247253   0.0238837   0.0259231
   H  -0.0113068  -0.0026847   0.0019206
   C  -0.0355933  -0.0121690  -0.0763340
   C  -0.0296766   0.0274978   0.1930887
   H   0.0200580  -0.0047860   0.0022872
   H  -0.0040116   0.0101607   0.0046828
   N   0.1503860  -0.0001084  -0.0791014
```

Toluene/Benzyl pair

```
29
Sum coordinate; displacements from reference geometry in Angstrom
   C  -0.0453047  -0.0665022   0.0074995
   C  -0.2159870  -0.2018836   0.0578958
   C  -0.0432062   0.0423468   0.0402706
   C   0.0711879   0.0360173  -0.0281272
   C   0.0739316   0.0836850  -0.0205123
   C   0.0946729   0.0425692  -0.0402950
   C   0.0396237   0.0484450  -0.0380253
   C  -0.0299393  -0.0671968   0.0234348
   C   0.0530677   0.2415047  -0.1263190
   C  -0.1159448  -0.0468155   0.1593404
   C  -0.0327586   0.1393928   0.0294750
   C   0.0102517  -0.1076503   0.0359158
   C   0.0344097  -0.0040998  -0.0930764
   C   0.0955142  -0.1370907  -0.0425820
   H  -0.0010190   0.0354696   0.0280518
   H   0.0265912   0.0524995  -0.0325262
   H   0.0277444   0.0467986  -0.0349499
   H   0.0136917   0.0160904   0.0345755
   H  -0.0707312   0.0011646   0.0666944
   H   0.4775380  -0.2307900   0.5638933
   H   0.0259807  -0.1127125   0.0322971
   H   0.0235886   0.0270299   0.0450994
   H   0.0066002  -0.0247871   0.0513902
   H  -0.0092786   0.0565359   0.0014112
   H   0.0274544  -0.0082525   0.0043769
   H   0.0125950   0.0295352  -0.0008754
   H  -0.0396858  -0.0387131   0.0281519
   H  -0.0040450   0.0051617   0.0814547
   H   0.0552513  -0.0258158   0.0004790
```

13.4 Difference coordinate

Phenol/Phenoxy pair

```
25
Difference coordinate; displacements from reference geometry in Angstrom
   C   0.2523482   0.1814985  -0.0266320
   C  -0.1345923  -0.0351714   0.1160370
   C   0.0526690   0.1017458   0.0734611
   C  -0.0188198   0.0166271  -0.0111508
   C   0.1403551   0.0815818  -0.0225771
   C  -0.0453636  -0.1224498  -0.1454567
   H   0.0057738   0.0352485   0.0412757
   H  -0.0041981   0.0377938   0.0394025
   H   0.0064238  -0.0475012  -0.0558313
   H   0.0632027  -0.0087488  -0.0094540
   H   0.0737586   0.0107872   0.0299486
   O  -0.2230828  -0.1481783   0.0184452
   H  -0.3846092   0.1509939   0.1630266
   O  -0.2756856   0.2208460  -0.0947931
   C   0.3016565  -0.2950453   0.1533460
   C  -0.1664251   0.0572747  -0.0196792
   C  -0.0186215  -0.1773115   0.1277277
   C  -0.0032505  -0.0176298   0.0078453
   C   0.1927596  -0.0301579  -0.0034048
   C   0.0439863   0.1302069  -0.1117507
   H  -0.0324057   0.0186876  -0.0227204
   H   0.0087157   0.0023029  -0.0217067
   H  -0.0262133   0.0253538  -0.0375624
   H   0.0382456   0.0006060  -0.0069858
   H   0.0331219   0.0390389  -0.0126714
```

Indole/Indolyl pair

```
31
Difference coordinate; displacements from reference geometry in Angstrom
   C  -0.0148102  -0.0945061   0.1342456
   C   0.0036511   0.0134018  -0.1318621
   C   0.0126868   0.0413322   0.0508229
   C  -0.0164245  -0.0512562   0.0005525
   C  -0.0179088  -0.0121234   0.0028494
   C   0.0099674   0.0460559  -0.0262077
   H   0.0095990  -0.0109750   0.0331199
   H  -0.0008451  -0.0157853   0.0117225
   H  -0.0155036  -0.0057096  -0.0237484
   H   0.0082448   0.0013543   0.0046695
   C   0.0125026   0.0879622  -0.2293801
   C  -0.0037269   0.2003013   0.2911454
   H   0.0038461   0.0249727   0.0569875
   H  -0.0203790   0.0166629   0.0417547
   N  -0.0022427  -0.2162383  -0.1143000
   H   0.0090120  -0.1618586  -0.4023447
   C   0.1552855   0.0222316   0.0944736
   C  -0.0050812  -0.0224375  -0.1582432
   C  -0.1161526  -0.0212565   0.0495986
   C   0.0855571   0.0248535   0.0081689
   C  -0.1050260   0.0036511  -0.0019610
   C  -0.2343963  -0.0374752  -0.0829244
   H   0.0208990   0.0105091   0.0014951
   H  -0.0036836   0.0247993   0.0387862
   H   0.0518846   0.0501187   0.0543981
   H  -0.0237267  -0.0056337   0.0040303
   C  -0.0746904  -0.0255360  -0.1601825
   C  -0.0622745   0.0577025   0.4051854
   H   0.0420906  -0.0100432   0.0047995
   H  -0.0084181   0.0213216   0.0098265
   N   0.3155763  -0.0002275  -0.1659896
```

Toluene/Benzyl pair

```
29
Difference coordinate; displacements from reference geometry in Angstrom
   C  -0.0544818  -0.0799731   0.0090186
   C  -0.2597380  -0.2427778   0.0696233
   C  -0.0519582   0.0509247   0.0484280
   C   0.0856080   0.0433131  -0.0338248
   C   0.0889074   0.1006365  -0.0246674
   C   0.1138502   0.0511922  -0.0484573
   C   0.0476501   0.0582581  -0.0457278
   C  -0.0360039  -0.0808084   0.0281818
   C   0.0638173   0.2904246  -0.1519067
   C  -0.1394310  -0.0562986   0.1916169
   C  -0.0393943   0.1676287   0.0354455
   C   0.0123284  -0.1294564   0.0431910
   C   0.0413798  -0.0049303  -0.1119302
   C   0.1148619  -0.1648602  -0.0512076
   H  -0.0012254   0.0426544   0.0337340
   H   0.0319776   0.0631340  -0.0391148
   H   0.0333644   0.0562782  -0.0420295
   H   0.0164651   0.0193497   0.0415792
   H  -0.0850587   0.0014005   0.0802042
   H  -0.5017362   0.0552165  -0.4070753
   H   0.0312434  -0.1355439   0.0388393
   H   0.0283667   0.0325052   0.0542349
   H   0.0079371  -0.0298080   0.0617999
   H  -0.0111581   0.0679880   0.0016970
   H   0.0330157  -0.0099241   0.0052635
   H   0.0151463   0.0355179  -0.0010527
   H  -0.0477247  -0.0465550   0.0338544
   H  -0.0048644   0.0062072   0.0979544
   H   0.0664432  -0.0310452   0.0005760
```

References

(S1) Bahmann, H.; Kaupp, M. Efficient Self-Consistent Implementation of Local Hybrid Functionals. *J. Chem. Theory Comput.* **2015**, *11*, 1540–1548.

(S2) Klawohn, S.; Bahmann, H.; Kaupp, M. Implementation of Molecular Gradients for Local Hybrid Density Functionals Using Seminumerical Integration Techniques. *J. Chem. Theory Comput.* **2016**, *12*, 4254–4262.

(S3) Maier, T. M.; Bahmann, H.; Kaupp, M. Efficient Semi-numerical Implementation of Global and Local Hybrid Functionals for Time-Dependent Density Functional Theory. *J. Chem. Theory Comput.* **2015**, *11*, 4226–4237.

(S4) Haasler, M.; Maier, T. M.; Grotjahn, R.; Gückel, S.; Arbuznikov, A. V.; Kaupp, M. A Local Hybrid Functional with Wide Applicability and Good Balance between (De)Localization and Left–Right Correlation. *J. Chem. Theory Comput.* **2020**, *16*, 5645–5657.

(S5) Weigend, F.; Ahlrichs, R. Balanced basis sets of split valence, triple zeta valence and quadruple zeta valence quality for H to Rn: Design and assessment of accuracy. *Phys. Chem. Chem. Phys.* **2005**, *7*, 3297–3305.

(S6) Balasubramani, S. G.; Chen, G. P.; Coriani, S.; Diedenhofen, M.; Frank, M. S.; Franzke, Y. J.; Furche, F.; Grotjahn, R.; Harding, M. E.; Hättig, C.; Hellweg, A.; Helmich-Paris, B.; Holzer, C.; Huniar, U.; Kaupp, M.; Marefat Khah, A.; Karbalaei Khani, S.; Müller, T.; Mack, F.; Nguyen, B. D.; Parker, S. M.; Perlt, E.; Rappoport, D.; Reiter, K.; Roy, S.; Rückert, M.; Schmitz, G.; Sierka, M.; Tapavicza, E.; Tew, D. P.; van Wüllen, C.; Voora, V. K.; Weigend, F.; Wodyński, A.; Yu, J. M. TURBOMOLE: Modular program suite for *ab initio* quantum-chemical and condensed-matter simulations. *J. Chem. Phys.* **2020**, *152*, 184107.

(S7) Weigend, F. Accurate Coulomb-fitting basis sets for H to Rn. *Phys. Chem. Chem. Phys.* **2006**, *8*, 1057.

(S8) Caldeweyher, E.; Ehlert, S.; Hansen, A.; Neugebauer, H.; Spicher, S.; Bannwarth, C.; Grimme, S. A generally applicable atomic-charge dependent London dispersion correction. *J. Chem. Phys.* **2019**, *150*, 154122.

(S9) Šrut, A.; Lear, B. J.; Krewald, V. The Marcus dimension: identifying the nuclear coordinate for electron transfer from ab initio calculations. *Chem. Sci.* **2023**, *14*, 9213–9225.

(S10) Mai, S.; Richter, M.; Heindl, M.; Menger, M. F. S. J.; Atkins, A.; Ruckenbauer, M.; Plasser, F.; Ibele, L. M.; Kropf, S.; Oppel, M.; Marquetand, P.; González, L. SHARC2.1: Surface Hopping Including Arbitrary Couplings — Program Package for Non-Adiabatic Dynamics. sharc-md.org, 2019; `http://sharc-md.org`.

(S11) Skone, J. H.; Soudackov, A. V.; Hammes-Schiffer, S. Calculation of Vibronic Couplings for Phenoxyl/Phenol and Benzyl/Toluene Self-Exchange Reactions: Implications for Proton-Coupled Electron Transfer Mechanisms. *J. Am. Chem. Soc.* **2006**, *128*, 16655–16663.

(S12) Sirjoosingh, A.; Hammes-Schiffer, S. Proton-coupled electron transfer versus hydrogen atom transfer: Generation of charge-localized diabatic states. *J. Phys. Chem. A* **2011**, *115*, 2367–2377.

(S13) Ludlow, M. K.; Skone, J. H.; Hammes-Schiffer, S. Substituent Effects on the Vibronic Coupling for the Phenoxyl/Phenol Self-Exchange Reaction. *J. Phys. Chem. B* **2008**, *112*, 336–343.

(S14) Neese, F.; *Technical Directorship* F. Wennmohs; *with contributions from* D. Aravena; Atanasov, M.; Auer, A. A.; Becker, U.; Bistoni, G.; Bykov, D.; Chilkuri, V. G.;

Datta, D.; Dutta, A. K.; Ehlert, S.; Ganyushin, D.; Garcia, M.; Guo, Y.; Hansen, A.; Helmich-Paris, B.; Huntington, L.; Izsák, R.; Kettner, M.; Kollmar, C.; Kossmann, S.; Krupička, M.; Lang, L.; Lechner, M.; Lenk, D.; Liakos, D. G.; Manganas, D.; Pantazis, D. A.; Papadopoulos, A.; Petrenko, T.; Pinski, P.; Pracht, P.; Reimann, C.; Retegan, M.; Riplinger, C.; Risthaus, T.; Roemelt, M.; Saitow, M.; Sandhöfer, B.; Schapiro, I.; Sen, A.; Sivalingam, K.; de Souza, B.; Stoychev, G.; Van den Heuvel, W.; Wezisla, B.; *and with contributions from collaborators* M. Kállay; Grimme, S.; Valeev, E.; Chan, G.; Pittner, J.; Brehm, M.; Goerigk, L.; Åsgeirsson, V.; Ungur, L. ORCA version 5.0.4, an *ab initio*, DFT and semiempirical SCF-MO package. 2023; `https://orcaforum.kofo.mpg.de/`.

(S15) Neese, F. Software update: The ORCA program system – Version 5.0. *WIREs Comput. Mol. Sci.* **2022**, *12*, e1606.

(S16) Angeli, C.; Cimiraglia, R.; Evangelisti, S.; Leininger, T.; Malrieu, J.-P. Introduction of n-electron valence states for multireference perturbation theory. *J. Chem. Phys.* **2001**, *114*, 10252–10264.

(S17) Angeli, C.; Cimiraglia, R.; Malrieu, J.-P. N-electron valence state perturbation theory: a fast implementation of the strongly contracted variant. *Chem. Phys. Lett.* **2001**, *350*, 297–305.

(S18) Angeli, C.; Cimiraglia, R.; Malrieu, J.-P. n-electron valence state perturbation theory: A spinless formulation and an efficient implementation of the strongly contracted and of the partially contracted variants. *J. Chem. Phys.* **2002**, *117*, 9138–9153.

(S19) Weigend, F. A fully direct RI-HF algorithm: Implementation, optimised auxiliary basis sets, demonstration of accuracy and efficiency. *Phys. Chem. Chem. Phys.* **2002**, *4*, 4285–4291.

(S20) Inagaki, T.; Yamamoto, T.; Kato, S. Proton-coupled electron transfer of the phe-

noxyl/phenol couple: Effect of Hartree-Fock exchange on transition structures. *J. Comput. Chem.* **2011**, *32*, 3081–3091.
